# *In vitro* characterization of the yeast DEAH/RHA RNA helicase Dhr1

**DOI:** 10.1016/j.jbc.2025.108366

**Published:** 2025-02-28

**Authors:** Ran Lin, Ezzeddine Elmir, Madison J. Reynolds, Arlen W. Johnson

**Affiliations:** 1 Department of Molecular Biosciences, The University of Texas at Austin Austin, Texas, USA; 2 Department of Radiation Oncology, Medical College of Wisconsin, Milwaukee, Wisconsin, USA; 3 Chicago-Kent College of Law, Chicago, Illinois, USA

**Keywords:** ribosome assembly, RNA helicase, DHR1, UTP14, SSU processome

## Abstract

In eukaryotic ribosome biogenesis, the small subunit (SSU) processome is a metastable intermediate in the assembly of the small (40S) subunit. In the SSU processome, the ribosomal RNA domains are splayed open by the intervention of assembly factors as well as U3 snoRNA. A critical step during the transition from the SSU processome to the nearly mature pre-40S particle is the removal of the U3 snoRNA to allow the formation of the central pseudoknot, a universally conserved structure which connects all domains of the subunit and contributes to its dynamic nature during translation. We previously identified the DEAH/RHA RNA helicase Dhr1 as the enzyme responsible for displacing the U3 snoRNA and the SSU processome factor Utp14 as an activator of Dhr1. Here, we have utilized biochemical and yeast genetic methods to further characterize Dhr1. We show that the N terminus as well as an internal loop within the RecA2 domain are autoinhibitory. We found that Utp14 can activate the ATPase activity of Dhr1 lacking the autoinhibitory N-terminal loop but not full-length Dhr1. We considered the possibility that Utp14 activates Dhr1 by relieving the autoinhibition of the loop within the RecA2 domain. However, our results are more consistent with Utp14 activating Dhr1 by binding to the surface of the RecA1 and RecA2 domains rather than displacing the inhibitory loop. This position of Utp14 is distinct from how G-patch proteins activate other DEXH/RHA helicases and is consistent with our previous conclusion that Utp14 is not a canonical G-patch protein.

Ribosomes are ribonucleoprotein machines that synthesize all proteins in living organisms. The two subunits of the ribosome have distinct functions: the large subunit catalyzes peptide bond formation while the small subunit (SSU) decodes messenger RNAs. Ribosome assembly is a complex process and the correct assembly of ribosomes is critical for high-fidelity gene expression. Assembly is initiated by the transcription of the ribosomal DNA repeats by RNA polymerase I. In yeast, RNA polymerase I synthesizes a 35S primary transcript that contains three rRNAs, 18S of the SSU, and 5.8S and 25S of the large subunit, flanked by external transcribed spacers and interrupted by two internal transcribed spacers (1). This precursor RNA undergoes cotranscriptional folding, processing, and modification so that the assembly and initial maturation of both subunits occur within the nucleolus. During processing, the spacer sequences are removed by endonucleases and exonucleases during ribosome assembly (2). Further maturation occurs in the nucleus and nuclear export to the cytoplasm enables the final stages of assembly and quality control (1, 3, 4).

A critical and conserved step in SSU biogenesis is the assembly of the SSU processome (also referred to as the 90S preribosome), a large complex of pre-rRNA, assembly factors, and a subset of ribosomal proteins (5, 6, 7, 8). The transition from the SSU processome to the mature 40S proceeds through multiple intermediates (3, 9, 10) and involves rRNA and protein rearrangements that result in compaction of the overall assembly. A critical element of the mature 40S subunit is the central pseudoknot (CPK), an RNA fold that organizes the overall architecture of the SSU and connects all four domains of the 18S ribosomal RNA (rRNA) (11). Formation of the CPK involves base pairing between disparate regions of the pre-rRNA: helix 1 forms between nucleotides 4 to 8 and 15 to 20 to generate a short stem loop while helix 2 of the CPK forms between nucleotides 10 to 14 within the loop formed by helix 1 and 1140 to 1144. The initial folding of 18S rRNA is guided, in part, by the U3 small nucleolar RNA, which base-pairs with the pre-rRNA in such a way as to preclude premature formation of the CPK, while bringing the distal nucleotides 1140 to 1144 into proximity for formation of the CPK ((5, 12, 13)). Thus, unwinding the U3 from the pre-rRNA to allow the folding of the CPK is an essential step in assembling the SSU. The DEAH helicase Dhr1 from *Saccharomyces cerevisiae*, also identified as Ecm16, is an essential 145 kDa protein that we previously reported and is required for unwinding U3 from 18S rRNA (14). Dhr1 is a member of the SF2 superfamily and is classified as a DEAH/RHA enzyme based on sequence similarity to other known RNA helicases. These enzymes typically display processive 3′ to 5′ unwinding activity (15, 16, 17). We previously used *in vivo* protein-RNA crosslinking to show that Dhr1 binds to U3 snoRNA, primarily nucleotides 22 to 63 of the BoxA region and the 5′ hinge (14). This crosslinking analysis placed Dhr1 on U3 snoRNA immediately downstream of the U3:18S heteroduplexes that are formed in the SSU processome, in position to disrupt this duplex if Dhr1 were to translocate in the 3′ to 5′ direction. We also showed that a catalytic mutant of Dhr1, Dhr1-K420A, failed to release U3 RNA *in vivo* (14), and was defective in unwinding *in vitro* (18). Lastly, recent structural studies of transition intermediates of the SSU processome reveal Dhr1 positioned adjacent to the U3:18S duplexes before their unwinding in the Dis-C intermediate (9, 19). These results identify the native substrate of Dhr1 to be the U3:18S duplexes. A similar conclusion has been reached for DHX37, the mammalian homolog of Dhr1 (20).

Since RNA conformational rearrangements driven by helicases may be irreversible, it is important to regulate the timing of such rearrangements. Enzymes of the DEAH/RHA helicase family, to which Dhr1 belongs, are commonly regulated by G-patch proteins (21, 22, 23, 24). Although Dhr1 does not appear to use a canonical G-patch protein, we previously identified the assembly factor Utp14 as an interactor of Dhr1 that activates its unwinding activity *in vivo* and *in vitro* (14, 25). Utp14 is a large 103 kDa protein predicted to be almost entirely unstructured. A previous genetic screen pinpointed a short, highly conserved sequence within Utp14 that was necessary to activate Dhr1(25). Biochemical analysis of mouse DHX37 and Utp14 revealed that this sequence of Utp14 was sufficient to enhance the RNA binding and ATPase activity of DHX37 (26). However, how the activation sequence of Utp14 interacts with Dhr1 to promote its activation is still unclear.

In this work, we have utilized biochemical and yeast genetic methods to characterize the self-regulation of Dhr1. We show that Dhr1 is autoinhibited by its N terminus as well as by an internal loop within the RecA2 domain of Dhr1. Our investigation here also revealed that ATPase activity showed that the Utp14 fragments do not release Dhr1 autoinhibition by interacting with the loop. Instead, they likely bind to the surface of Dhr1 to activate its enzyme activity. This finding supports our earlier hypothesis that Utp14 activates Dhr1 through a noncanonical mechanism.

## Results

### Autoinhibition of Dhr1

**Figure 1:**
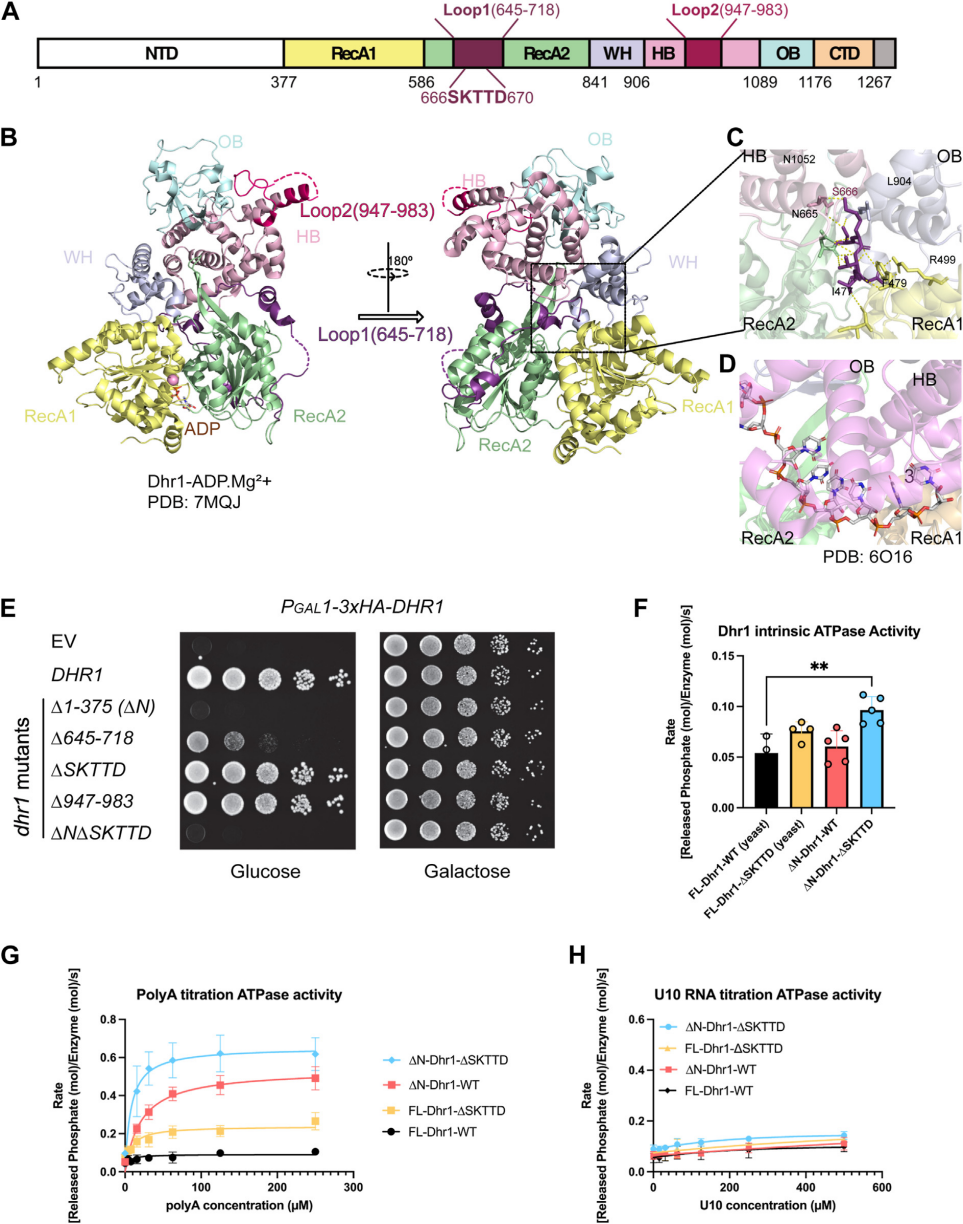
**Identifying autoinhibitory elements of Dhr1**. *A*, a schematic of Dhr1 domain organization. NTD, N-terminus domain; RecA1, RecA-like domain 1; RecA2, RecA-like domain 2; WH; winged helix domain; HB, helical-bundle domain; OB, oligonucleotide-binding fold domain; CTD, C-terminus domain. Loop regions are highlighted with *purple* (Loop 1, 645–718) and rose (Loop 2, 947–983). *B*, the structure of yeast Dhr1^377-1174^ with ADP•Mg^2+^ (PDB: 7MQJ). Dhr1 is shown in cartoon format and colored according to the scheme in panel *A*. Loops are shown as *dashed lines*. The structure is also demonstrated by rotating 180° to view the loops better. *C*, polar contacts between the amino acid residues SKTTD within loop 1 and other residues in yeast Dhr1. Amino acids 666 to 670 are shown in stick representation and colored with *purple*. Interacting residues are also shown as *sticks*. *D*, DHX37 structure with U10 RNA (PDB: 6O16), aligned with 7MQJ *via* RecA2 domain. *E*, Dhr1 complementation. *P*_*GAL1*_*-DHR1* (AJY3711) cells containing either empty vector (EV), WT, or vectors encoding the indicated alleles of *DHR1* spotted on SD-Leu containing glucose (*left*) or galactose (*right*) grown for 48 h at 30 °C. *F*, intrinsic ATPase activity of Dhr1 and its mutant variants. ATPase assays were performed multiple times (N ≥ 4). Individual data points are shown, and the error bars represent the standard error of the means. In all assays, 0.5 μM of the enzyme was used. *G*, polyA-stimulated ATPase activity of Dhr1 and its mutant variants. ATPase activity was measured at various polyA concentrations (0–250 μM, nucleotide concentration). Assays were performed in triplicate. Data points represent the mean of the triplicates, and the error bars represent the standard error of the means. In all assays, 0.5 μM of the enzyme was used. *H*, oligo RNA-stimulated ATPase activity of FL-Dhr1-WT, FL-Dhr1-ΔSKTTD, ΔN-Dhr1, and ΔN-Dhr1-ΔSKTTD was measured at various oligo U10 concentrations (0–500 μM, nucleotide concentration). Assays were performed in triplicate. Data points represent the mean of the triplicates, and the error bars represent the standard error of the means. In all assays, 0.5 μM of the enzyme was used. PDB, Protein Data Bank.

Dhr1 contains seven domains (Fig. 1*A*). High-resolution structures of yeast Dhr1, obtained by X-ray crystallography, have resolved six domains, including the conserved catalytical core of Dhr1 (Fig. 1*B*). Structures of Dhr1 homologs from different species have also been solved (9, 19, 26, 27). Although the N terminus (amino acids (aa) 1 to 376) of yeast Dhr1 has not been resolved by X-ray crystallography and is predicted by AlphaFold to be unstructured (https://alphafold.ebi.ac.uk/entry/Q04217), portions of the N terminus of Dhr1 have been observed in processome structures by cryo-EM. Dhr1 appears to be recruited to the processome following cleavage at site A1 (28). The N terminus of Dhr1 threads through the post-A1 complex and interacts with the GTPase Bms1 and the methyltransferase Dim1 as well as 18S rRNA and U3 snoRNA. Dhr1 persists in the Dis-C complex (Protein Data Bank: 6ZQG) (9)which has shed many factors of the full 90S complex but retains U3 bound to 18S (Fig. S1). Thus, the N terminus is likely important for the recruitment and stable interaction of Dhr1 with the processome and subsequent intermediates.

Dhr1 differs from other DEXH RNA helicases by having two insertions within the domains that comprise the core of the enzyme. One is an insertion within the RecA2 domain (aa 645–718, loop 1) and a second is an insertion in the helical-bundle (HB) domain (aa 945–983, loop 2). A recent crystal structure of yeast Dhr1 (19) revealed that loop 1 occupies the cleft between the RecA, HB, and oligonucleotide-binding (OB) domains (Fig. 1*B*). As this is the substrate channel (Fig. 1*D*), the RecA2 domain insertion is positioned to sterically block RNA binding (comparing Fig. 1, *C* and *D*), leading to the suggestion that this insertion acts as an autoinhibitory element (19). Close inspection of the Dhr1 structure identified five residues within the putative autoinhibitory loop, Ser-Lys-Thr-Thr-Asp (SKTTD, aa 666–670), that contact the domains of Dhr1 and are likely responsible for stabilizing the loop within the substrate channel (Fig. 1*C*). Ser666 interacts with Asn1052 of the HB domain; Lys667 interacts with Asn1052 and Leu904 of the OB domain and Thr668 interacts with Arg499, Phe479, and Ile477 of the RecA1 domain. Although the loop 1 insertion within the RecA2 domain is universally conserved in eukaryotes, the SKTTD motif appears to be found only among Ascomycota (Fig. S2*A*). Loop 1 was not resolved in an earlier crystal structure of yeast Dhr1 (27), suggesting that loop 1 is easily displaced from the RNA channel and its absence from structures does not necessarily imply that it does not provide an autoinhibitory function. Loop 1 was also not observed in the crystal structure of mouse DHX37. Thus, it remains an open question whether or not loop 1 of Dhr1 from other species is also autoinhibitory. Crystal structures of yeast Dhr1 have not revealed a potential function for loop 2.

To begin to determine the function of these insertions in Dhr1, we deleted each loop and the unstructured N terminus individually and tested the ability of the mutants to complement the loss of *DHR1*. Deleting the N terminus was lethal (Fig. 1*E*). Deleting loop 1 within the RecA2 domain severely impaired Dhr1 function *in vivo*, whereas deletion of loop 2 in the OB domain had no noticeable effect on its function (Fig. 1*E*). To narrow down the region of loop 1 that is critical for its function, we made nested deletions of loop 1 and tested their function *in vivo* (Fig. S2*B*). Deletion of SKTTD on Dhr1 had no obvious impact on *DHR1* function, while deletion of 35 or fewer residues of loop 1 only modestly affected function (Fig. S2*B*). In contrast, deletion of 45 residues or the entire loop 1 (Δ645–718, Δ74) strongly impaired function, possibly, because these deletions perturb the overall structure of the RecA2 domain. To confirm that the phenotypes of mutant Dhr1 variants were not due to differences in expression levels, vectors containing WT or mutant Dhr1 were transformed into a yeast strain expressing GFP-tagged Dhr1 to distinguish the vector-borne Dhr1 from the endogenous protein. Western blot analysis (Fig. S2*C*) demonstrated that all mutant Dhr1 variants were expressed at levels comparable to or greater than the endogenous GFP-tagged Dhr1.

Because deletion of the N terminus and RecA2 domain insertions differentially affected function *in vivo*, we sought to understand how they impacted Dhr1 function *in vitro*. We expressed and purified four different versions of Dhr1, full-length Dhr1 (FL-Dhr1-WT), Δ1 to 375-Dhr1 (ΔN-Dhr1-WT), Dhr1 deleted of the five residues SKTTD within loop 1 (FL-Dhr1-ΔSKTTD), and Dhr1 combining the N terminal and internal deletions (ΔN-Dhr1-ΔSKTTD) and measured their ATPase activity. All four versions showed low intrinsic ATPase activity, in the absence of added RNA (Fig. 1*F*). However, the mutant protein deleted of both the N terminus and the SKTTD motif showed a slight but statistically significant increase in the Dhr1 intrinsic ATPase activity. These data indicate that the presence of the N terminus and the SKTTD limits the intrinsic ATPase activity of Dhr1.

As DEAH/RHA helicases typically display RNA-dependent ATPase activity, we also performed ATPase assays in the presence of RNA. The ATPase activity of all versions of Dhr1 was enhanced by the addition of polyA (Fig. 1*G*, Table 1). Deletion of either the N terminus (ΔN-Dhr1-WT) or SKTTD (FL-Dhr1-ΔSKTTD) strongly enhanced the RNA-dependent ATPase activity of Dhr1 over that of the full-length protein, and deletion of both the N terminus and SKTTD motif (ΔN-Dhr1-ΔSKTTD) showed an additive effect. Thus, both the N terminus and the SKTTD motif are autoinhibitory. Previous biochemical studies of N terminally truncated DHX37, the mouse homolog of Dhr1, employed oligonucleotides rather than polyA for stimulating RNA-dependent ATPase activity (26). Unlike DHX37, we found that FL-Dhr1-WT and the mutant variants were minimally activated by oligo U10 RNA (Fig. 1*H*, Table 1). *In vivo*, the N terminus may be responsible for inhibiting the promiscuous activity of the enzyme before its recruitment to the processome. However, once engaged with the processome, the N terminus is extended away from the helicase core (Fig. S1), leaving the SKTTD motif as a critical regulatory element for the activity of Dhr1.

**Table 1:** Kinetic parameters of ATPase activity of WT and mutant Dhr1

Intrinsic ATPase activity			
Enzyme	Initial [E] (μM)	Initial rate (s^-1^)	
FL-Dhr1-WT	0.5	0.05 ± 0.01	
FL-Dhr1-ΔSKTTD	0.5	0.08 ± 0.01	
ΔN-Dhr1-WT	0.5	0.06 ± 0.02	
ΔN-Dhr1-ΔSKTTD	0.5	0.10 ± 0.01	


ATPase activity (substrate: polyA)			
Enzyme	Initial [E] (μM)	*Kapp* (polyA) (μM)	*V*_max_(s^-1^)
FL-Dhr1-WT	0.5	N/D	0.12 ± 0.01
FL-Dhr1-ΔSKTTD	0.5	11.28 ± 2.97	0.28 ± 0.03
ΔN-Dhr1-WT	0.5	23.76 ± 3.32	0.55 ± 0.05
ΔN-Dhr1-ΔSKTTD	0.5	9.00 ± 3.00	0.67 ± 0.09


ATPase activity (substrate: U10)			
Enzyme	Initial [E] (μM)	*Kapp* (U10) (μM)	*V*_max_ (s^-1^)
FL-Dhr1-WT	0.5	23.31 ± 11.98	0.10 ± 0.02
FL-Dhr1-ΔSKTTD	0.5	13.43 ± 3.55	0.13 ± 0.02
ΔN-Dhr1-WT	0.5	7.06 ± 2.13	0.09 ± 0.01
ΔN-Dhr1-ΔSKTTD	0.5	10.06 ± 1.36	0.13 ± 0.01

Table 1. ATPase assay parameters of the indicated variants of Dhr1. Assays were performed in triplicates. The mean of the triplicates and the standard error deviation are shown. ATPase parameters of different Dhr1s with the presence of the RNA substrates (polyA or U10 RNA) are also shown. The kinetic values were generated using the GraphPad Prism software following Michaelis–Menten kinetics. Initial enzyme concentration [E] was plotted. *Kapp*: K apparent. *Vmax*: maximum reaction rate.

To ask directly if loop 1 prevents RNA binding, we performed fluorescence polarization binding assays on ΔN-Dhr1-WT and ΔN-Dhr1-ΔSKTTD (Fig. S3). As expected, the removal of the SKTTD decreased the Kd for oligo U20 about five-fold, from 0.30 ± 0.02 μM to 0.06 ± 0.01 μM (Fig. S3*D*). This was comparable to the change in *Kapp* measured for polyA-stimulated ATPase activity from 23.76 ± 3.32 μM to 9.00 ± 2.99 μM. Thus, removing SKTTD enhances RNA binding, which is consistent with the proposal that loop 1 inhibits Dhr1 activity by sterically blocking RNA binding. Previous work with mouse DHX37 showed that the RNA binding affinity of Dhr1 was reduced significantly in the presence of ADP or AMP-purine nucleoside phosphorylase (PNP). In contrast, we did not observe any significant change in RNA binding by yeast Dhr1 upon adding ADP or AMP-PNP (Fig. S3, *B* and *C*).

### Hyperactive Dhr1-ΔSKTTD suppresses Utp14 activation domain mutants

**Figure 2:**
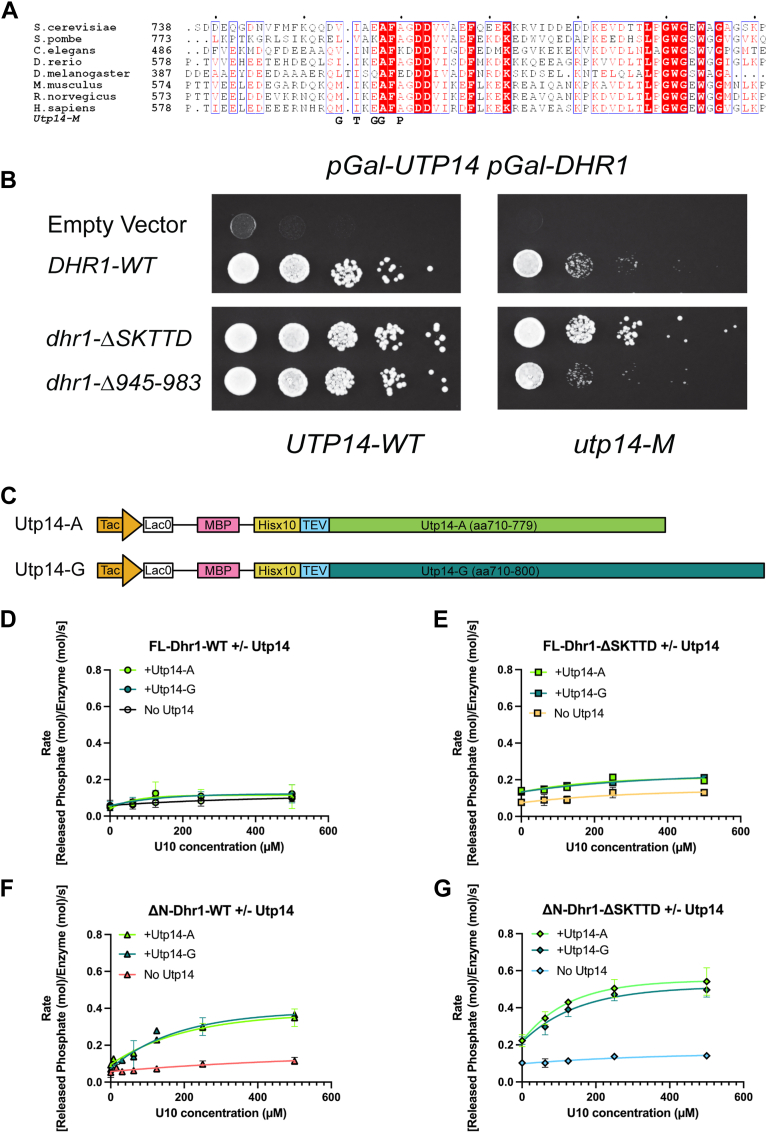
**Short motifs of Utp14 activate Dhr1 and Dhr1ΔSKTTD ATPase activity**. *A*, multisequence alignment of amino acids 738 to 801 from yeast Utp14 and its analogs, encompassing the activation domain of Utp14. Mutations in Utp14, previously identified as adversely affecting the Dhr1 function (25), that were combined to generate mutant Utp14 (Utp14-M) are shown at the *bottom*. The sequences have been aligned using T-coffee (35) subsequently adjusted manually and then output obtained using ESPript 3.0 (36). Aligned sequences: protein, organism (access No.): UTP14, *Saccharomyces cerevisiae* S288C (NP_013617.1); UTP14, *Schizosaccharomyces pombe* (NP_593375.1); UTP14 (*Danio rerio*, XP_005165685.1); CG12301, *Drosophila melanogaster* (NP_593520.1); UTP14A, *Mus musculus* (NP_082552.1); UTP14A, *Rattus norvegicus* (NP_001014135.2); UTP14A, *Homo sapiens* (KAI4000946.1). *B*, *P*_*GAL1*_*-UTP14 P*_*GAL1*_*-DHR1* (AJY4605) cells containing either WT *UTP14* (UTP14-WT) (left) or Mutant *UTP14* (UTP14-M) (*right*) and WT *DHR1* or vectors encoding the indicated mutant alleles were spotted onto SD-Leu containing media and grown for 48 h at 30 °C. *C*, a schematic for Utp14-A and Utp14-G expression constructs. *D*, oligo RNA-stimulated ATPase activity of FL-Dhr1-WT or with the presence of Utp14-A or Utp14-G. ATPase activities were measured at various oligo U10 concentrations (0–500 μM, concentration of single uridine). In all assays, 0.5 μM of the Dhr1 was used, and 2 μM of Utp14-A or Utp14-G were used. *E*, oligo RNA-stimulated ATPase activity of FL-Dhr1-ΔSKTTD or with the presence of Utp14-A or Utp14-G. *F*, oligo RNA-stimulated ATPase activity of ΔN-Dhr1-WT or with the presence of Utp14-A or Utp14-G. *G*, oligo RNA-stimulated ATPase activity of ΔN-Dhr1-ΔSKTTD or with the presence of Utp14-A or Utp14-G. For panels *D* through *G* assays were performed in triplicates. Data points represent the mean of the triplicates, and the error bars represent the standard error of the means.

Our previous studies showed that Dhr1 is activated by the ribosome assembly factor Utp14 (14, 25). Utp14 is predicted to be almost entirely devoid of structure, and we have previously mapped interactions between Utp14 and pre-rRNA spanning disparate regions of the SSU processome, including direct interaction with Dhr1 and overlapping binding sites with Dhr1 on U3 snoRNA(29). We have proposed that Utp14 helps recruit Dhr1 to the U3-18S heteroduplex and activates it for unwinding. We previously narrowed down the region of Utp14 that interacts with Dhr1 to aa 565 to 813(14, 25). Within this region, we identified a highly conserved sequence (Fig. 2*A*) which, when mutated (Utp14-M), blunted the ability of Utp14 to activate Dhr1 *in vivo* and *in vitro*, suggesting that this region of Utp14 was necessary for activation of Dhr1(25). Segments of Utp14 have been resolved in structures of the SSU processome and subsequent intermediates, but the activation domain and its mechanism of activating Dhr1 remain to be revealed. Biochemical analysis of murine DHX37 has demonstrated that the corresponding region of mouse Utp14 was sufficient to activate the RNA-dependent ATPase activity of DHX37 (26). In contrast, we did not observe a similar *in vitro* stimulation of full-length Dhr1 ATPase activity by Utp14 in our previous work (14).

Our results above, showing that Dhr1 employs autoinhibition, raise the question of whether Utp14 activates yeast Dhr1 by relieving its autoinhibition. One possibility is that Utp14 interacts directly with the autoinhibitory loop to expose the RNA binding cleft and release autoinhibition. We first tested this idea genetically, asking if *DHR1* lacking the autoinhibitory loop could bypass the need for activation by Utp14 using a *UTP14* with a mutant activation domain (*utp14-M*) (Figs. 2*B*, S4). Remarkably, all versions of loop1 deletion of Dhr1 suppressed *utp14-M* to some extent (Fig. S4), with *dhr1-ΔSKTTD* being the strongest suppressor. These results are consistent with the idea that Utp14 displaces the autoinhibitory loop (however, see below).

To further test the hypothesis that Utp14 activates Dhr1 by relieving its autoinhibition, we used ATPase assays *in vitro*. We purified two fragments of Utp14, Utp14-A (aa 710–779), and Utp14-G (aa 710–800), as fusions to maltose binding protein (Fig. 2*C*). We designed Utp14-A to span the region encompassing the activation domain of Utp14. The corresponding fragment from mouse Utp14 has been described previously as UTP14A^min^ which was reported to be a minimal peptide sufficient to activate mouse DHX37(26). We also designed an extended version as Utp14-G (aa710–800) (Fig. 2*C*) which included a downstream conserved tract reminiscent of a G-patch (Fig. 2*A*) (25). We measured ATPase activities of four different versions of Dhr1 (full-length, ΔN, full-length ΔSKTTD, and ΔNΔSKTTD (Fig. 2, *C*–*G*) in the presence of Utp14-A or Utp14-G. All four versions of Dhr1 showed low intrinsic ATPase activity, in the absence of RNA or Utp14 (Fig. S5). Neither FL-Dhr1-WT nor ΔN-Dhr1-WT was activated by Utp14 fragments. However, FL-Dhr1ΔSKTTD and ΔN-Dhr1-ΔSKTTD showed a small but statistically significant increase in intrinsic ATPase activity in the presence of Utp14-A or Utp14-G fragments. Removal of both the N terminus and the SKTTD motif further increased the activation. This result indicates that Utp14 activates the intrinsic ATPase activity of Dhr1 lacking the autoinhibition loop but not intact Dhr1 and suggests that Utp14 activates Dhr1 by a mechanism different from displacing the autoinhibitory loop. Utp14-A and Utp14-G activated the intrinsic Dhr1 activity to a similar degree, suggesting both fragments bind Dhr1 with a similar affinity. As shown in Figure 1*G*, the ATPase activity of all versions of Dhr1 was enhanced by the addition of polyA. However, the addition of Utp14 fragments did not increase the RNA-dependent ATPase activity of Dhr1 (Figs. S6 and S7), which is consistent with our previous report (25). Since polyA is a mixture of different lengths of RNAs, we assumed that the long RNA oligos are contributing the most to accelerate the Dhr1 ATPase activity. Utp14 fragments will not enhance the activity anymore.

As shown in Figure 1*H*, Dhr1 ATPase activity was not significantly enhanced by RNA oligos no matter if the SKTTD motif was present or not. We next asked if Utp14 fragments could activate Dhr1 ATPase activity with the presence of RNA oligos. Neither Utp14-A nor Utp14-G enhanced the ATPase activity of FL-Dhr1-WT in the presence of RNA oligos (Fig. 2*D*). Mutant Dhr1 lacking the SKTTD was slightly activated by Utp14 fragments, but independent of RNA (Fig. 2*E*), reflecting the modest activation of the intrinsic ATPase activity of Dhr1 (Fig. S5*D*). However, ΔN-WT was significantly activated by Utp14 fragments with the increasing concentration of RNA, suggesting that Utp14 fragments enhance ΔN-WT activity by increasing RNA binding affinity to ΔN-WT (Fig. 2*F*). This result also suggests that the presence of the N terminus of Dhr1 blocks Utp14 binding, likely contributing to the difference reported for Utp14 activation of full-length yeast Dhr1 versus N terminally truncated DHX37 (14, 25, 26). Deleting the N terminus from Dhr1-ΔSKTTD resulted in increased activation by Utp14, presumably reflecting its stimulation of the intrinsic ATPase activity as well as enhanced RNA-dependent activation (Fig. 2*E*). These results show that Utp14 fragments can activate Dhr1 without the presence of SKTTD, strongly suggesting that Utp14 does not activate Dhr1 by binding directly to and displacing its autoinhibitory loop.

### Predicting the Utp14 binding site on Dhr1

**Figure 3:**
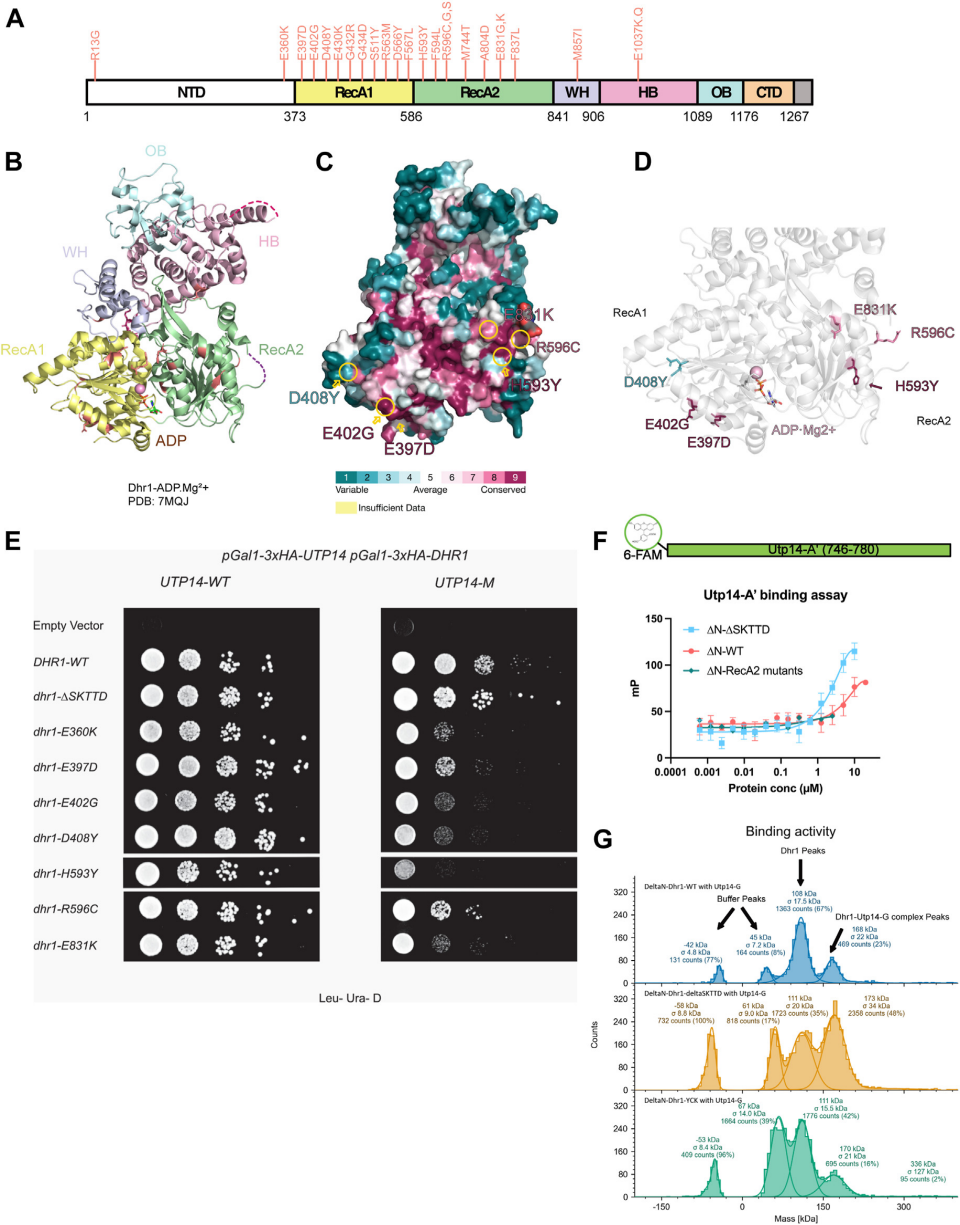
**Mapping Utp14 binding sites on Dhr1**. *A*, a cartoon of the primary structure of Dhr1 is shown. The domains and color codes follow the same rules as in Figure 1*A*. Mutations in *DHR1* that suppress *bud23*Δ are indicated in *salmon*. Numbering indicates amino acid residue of yeast Dhr1. *B*, the structure of yeast Dhr1^377-1174^ with ADP•Mg^2+^ (PDB: 7MQJ). Dhr1 is shown in cartoon format and mutants are colored as *salmon*. *C*, the surface map of Dhr1 (PDB: 7MQJ) is shown in Consurf-DB Analysis. Conservation level from 1 (most variable) to 9 (most conserved) of each residue is mapped to the surface. *D*, cartoon structure of RecA1 and RecA2. (PDB: 7MQJ, colored in *gray*). *bud23Δ* suppressing mutations mapping to the RecA1 and RecA2 surfaces of Dhr1 are shown in color using the coloring scheme for conservation. *E*, complementation of *dhr1* surface mutants. *P*_*GAL1*_*-DHR1* (AJY3711) cells containing either empty vector (EV), WT, or vectors encoding the indicated alleles of *DHR1* spotted on SD-Leu containing glucose (*left*) grown for 48 h at 30 °C. *F*, *in vitro* binding assay. Utp14 fragment (Utp14-A′, aa746–780) was tagged with 6-FAM on the N terminus. The binding activities of ΔN-Dhr1-WT and ΔN-Dhr1-ΔSKTTD were measured at various protein concentrations (0.000625–10 μM). The binding activity of ΔN-Dhr1-RecA2-mutants was measured at various protein concentrations (0.000625–2.5 μM). Assays were performed in triplicate. Data points represent the mean of the triplicates, and the error bars represent the standard error of the means. *G*, single-molecule binding assay by mass photometry. Mass photometry analysis of ΔN-Dhr1-WT or ΔN-Dhr1-WT or ΔN-Dhr1-YCK binding with UTP14-G fragment. PDB, Protein Data Bank.

The question remains: how does Utp14 interact with Dhr1 to activate its ATPase activity? Considering the high degree of conservation of the activation domain of Utp14, we anticipate that it should interact with a similarly conserved region of Dhr1. In our previous studies with the assembly factor Bud23, we identified multiple mutations within *UTP14* and *DHR1* that had the common effect of suppressing the growth defect of a *bud23* deletion mutant (25, 30, 31). These mutations in *UTP14* mapped to its activation domain and resulted in reduced activation of Dhr1, both *in vivo* and *in vitro* (25). The majority of the changes in Dhr1 were mapped to the RecA1 and RecA2 domains (Fig. 3, *A* and *B*). Some of these mutations, including R563M, D566Y, and F837L, were located in the interface of the two RecA domains (Fig. S8*B*), while E1037K was in the HB domain (Fig. S8*B*), which makes up part of the RNA binding cleft of Dhr1. Other suppressing mutations mapped to the surface of the RecA1 domain (E397D, E402G, and D408Y) or the surface of the RecA2 domain (H593Y, R596C, and E831K) (Fig. 3, *C* and *D*). We reasoned that those mutations in Dhr1 that defined its binding site with the activation domain of Utp14 should show synergistic negative genetic interaction when combined with mutations in Utp14 that reduced its activation of Dhr1. Therefore, we made pairwise combinations of *dhr1* mutations identified as suppressors of *bud23Δ* with the partially active *utp14-M* mutant.

Surprisingly, several of the mutations in the interface of the RecA domains suppressed the growth defect of the *utp14-M* mutant (Fig. S8*C*), as we had observed for *dhr1-ΔSKTTD* (Fig. 2*B*). *dhr1*-R563M and *dhr1*-D566Y showed near WT levels of growth, indicating that these mutations were able to nearly fully suppress *utp14*-*M*, bypassing the function of the Utp14 activation domain *in vivo*. Other mutations, such as *dhr1*-F837L suppressed poorly (Fig. S8*C*), or in the case of *dhr1-E1037K*, was synthetic lethal with *utp14-M* (Fig. S8*C*). In the structure of mouse DHX37, E880 (E1037 in yeast) is a critical residue in the RNA binding channel that makes interdomain hydrogen bonds to R304 and Y331 (R452 and F479 in yeast) to create the RNA binding channel (Protein Data Bank: 6O16). And, because R304 in DHX37 interacts directly with RNA, mutation of E880 is expected to negatively impact RNA binding of Dhr1.

Considering that mutations in the RecA domain interface could suppress *utp14-M*, we asked if they also altered the RNA-dependent ATPase activity of Dhr1. ΔN-Dhr1-F837L displayed a higher *V*_max_ (0.12  ± 0.01 s^-1^) compared to ΔN-Dhr1-WT (0.08  ± 0.01) indicating that it is a hyperactive mutant (Fig. S9). The *V*_max_ of both ΔN-Dhr1-D566Y (0.10  ± 0.01 s^-1^) and ΔN-Dhr1-R563M (0.10  ± 0.03 s^-1^) were also slightly higher than ΔN-Dhr1-WT but not as high as ΔN-Dhr1-F837L. These mutations F837L, D566Y, and R563M are located between the two RecA domains (Fig. S8) where they could affect the intrinsic dynamics of domain movement in cycles of ATP binding and hydrolysis. In contrast to the RecA interface mutations, ΔN-Dhr1-E1037K displayed markedly reduced ATPase activity (Fig. S9) and low affinity for polyA.

Because the RecA domain interface mutants did not show negative genetic interaction with mutant *utp14*, our criterion for potential Utp14 interaction mutants, we turned to the mutations on the surface of the RecA domains (E397D, E402G, D408Y, H593Y, R596C, and E831K) (Fig. 3*C*). We also included E360K, which affects a residue adjacent to the RecA1 domain but is not resolved in current structures. All individual mutations fully complemented the loss of *DHR1*. Encouragingly, all were synthetic sick with mutant *Utp14-M* with the exception of R596C (Fig. 3*E*).

To test directly if mutations on the surface of the RecA1 and RecA2 domains of Dhr1 affect Utp14 binding, we turned to *in vitro* binding assays. Because we have previously found that with Utp14 we could enhance the biochemical defect of activating Dhr1 by combining multiple single suppressing mutations, we used a similar strategy with Dhr1. We combined mutations on the surface of RecA1 (E360K, E397D, E402G, D408Y: *dhr1-KDGY*) or RecA2 (H593Y, R596C, and E831K: *dhr1-YCK*), or both (*dhr1-KDGYYCK*). While the ΔN-Dhr1-YCK (RecA2 surface) protein behaved well, the ΔN-Dhr1-KDGY (RecA1 surface mutants) was not soluble and could not be purified. We performed fluorescence polarization binding assays with ΔN-Dhr1-WT, ΔN-Dhr1-YCK (RecA2 surface) and ΔN-Dhr1-ΔSKTTD for comparison. We found that ΔN-Dhr1-YCK displayed reduced binding whereas the removal of SKTTD enhanced Utp14 fragment binding compared to WT (Fig. 3*F*). However, in these binding assays, we were not able to reach saturation, and consequently, we were unable to calculate Kds.

As an alternative method to measure the binding between Dhr1 variants and Utp14, we used single-molecule mass photometry (MP) (32). Calibration was done using 10 nM of β-amylase (monomer 56 kD, dimer 112 kD, and tetramer 224 kD) and 10 nM of thyroglobulin (bovine) (monomer 335 kD, dimer 670 kD, trimer 1005 kD, and tetramer 1340 kD) (Fig. S11*A*, *B*). As shown, the components generated symmetric peaks at ± 50 kD, which indicated the buffer peaks. The buffer peaks will merge into protein peaks with a similar molecular weight (like Utp14-G, Fig. S11*F*). The histograms for apo Dhr1s and Utp14-G are shown in Fig. S11, *C*–*F*. As shown in Figure 3*G*, the mixture of ΔN-Dhr1-WT and Utp14-G at a 1:1 ratio generated 23% complex, at the molecular weight of 168 kD ± 22 kD. Similar reactions but using ΔN-Dhr1-YCK and ΔN-Dhr1-ΔSKTTD, yielded 16% complex at 170 kD ± 21 kD and yielded 48% complex, at 173 kD ± 34 kD, respectively. These results are consistent with the peptide binding assay, confirming that the RecA2 surface mutant has reduced binding with Utp14-G while deletion of SKTTD enhances the binding of Utp14.

## Discussion

Based on a combination of genetic and biochemical results, we propose that Utp14 binds across the RecA domains in a manner that optimizes the dynamics of the two domains during cycles of ATP binding and hydrolysis. This conclusion is based, in part, on genetic results in which we found synthetic negative genetic interaction between mutations in *UTP14* that reduce its ability to activate Dhr1 and mutations in *DHR1*, mapping to the conserved surfaces of RecA 1 and RecA2 domains. It is also based on physical evidence showing that Dhr1, in which residues on the surface of RecA2 are mutated, has reduced affinity for Utp14. Unfortunately, we were unable to determine the effects of similar mutations on the surface of RecA1. Interestingly, we found that deletion of the autoinhibitory loop 1, which inserts into the RNA binding cleft, enhanced Utp14 binding. We suggest that when the autoinhibitory loop is engaged in the RNA cleft, the relative positioning of the two RecA domains is suboptimal for Utp14 binding across these domains. This situation is relieved by deleting Loop1, allowing the two RecA domains to adopt favorable positioning for Utp14 binding.

**Figure 4:**
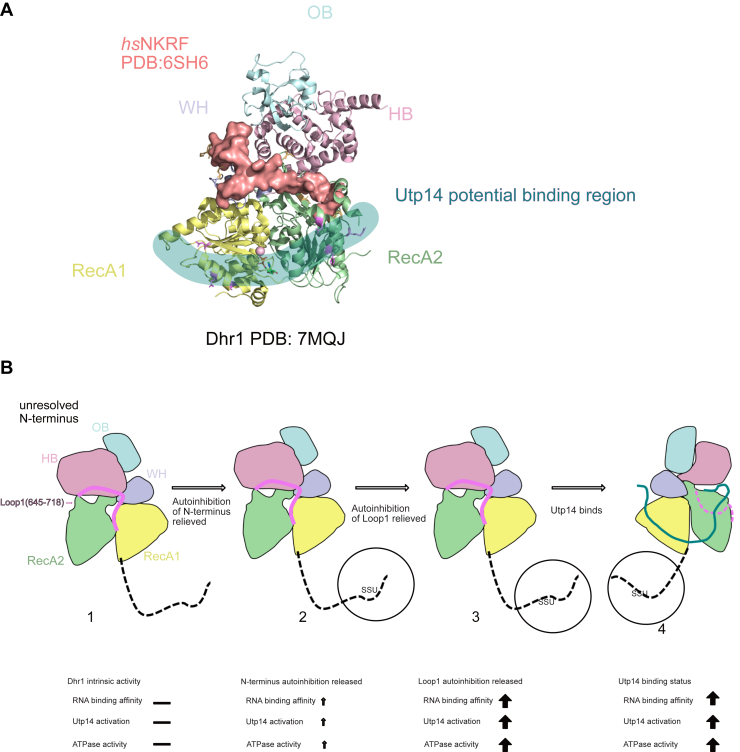
**Mechanism of regulation of Dhr1 helicase**. *A*, the G-patch from NKRF (PDB: 6SH6) docked into the corresponding position in Dhr1 structure (PDB: 7MQJ). Dhr1 is shown in cartoon mode, and NKRF is shown in surface mode (colored in *salmon*). Residues of Dhr1 proposed to bind with Utp14 are colored in *pink*. Proposed Utp14 binding surface on RecA1 and RecA2 domains is highlighted with *blue-green*. *B*, model of the mechanism of Dhr1 helicase action regulation by releasing its autoinhibition and interaction with Utp14. Intact protein activity is shown as “−”, higher activity is shown as “”, highest activity is shown as “”. From left to right: 1. intact Dhr1 RNA binding activity, Utp14 activation (*in vitro*) and ATPase activity are at baseline states. 2. From Figure 1*G* polyA titration activity, N terminus deletion shows a higher ATPase activity associated with increasing with RNA concentration increasing; this indicates RNA binding affinity is increased by N terminus removal. From Figure 2, *D* and *F*, Utp14 fragments also increase the ATPase activity of N terminus deletion but not the intact Dhr1; this indicates the removal of N terminus increases RNA binding affinity, Utp14 activation (*in vitro*), and ATPase activity. 3. Under the condition of removal the loop 1 and the N terminus: from Figure S3*A*, RNA binding activity shows removal the loop 1 increases the RNA binding affinity, Fig. S5, *C* and *D* show the Utp14 increases the intrinsic ATPase activity of ΔN-ΔSKTTD (*in vitro*). Figure 2, *F* and *G* also show the increase of RNA dependent activity. 4. After both autoinhibition released, Utp14 binds Dhr1s in a catalytical way to process helicase activity. PDB, Protein Data Bank.

Many DEAH/RHA helicases are activated by G-patch proteins (24) Recent crystal structures of the splicing helicase Prp2 in complex with its G-patch protein Spp2 from *Chaetomium thermophilum* and human DHX15 with its G-patch protein NKRF have revealed a common mode of interaction. In these complexes, the G-patch lies across the back side of the helicase, with respect to the RNA binding cleft, stretching from the Winged-helix domain to the RecA2 domain (Fig. 4*A*) (24). Thus, our proposed placement of the activation domain of Utp14 laying across the RecA1 and RecA2 domains would represent a mode of activation that is distinct from how canonical G-patch proteins activate their cognate RNA helicases.

Figure 4*B* summarizes our results about autoinhibition of Dhr1 and its activation by Utp14. Prior to binding to the SSU processome, Dhr1 is maintained in an autoinhibited state by the N terminus and loop 1 within the RecA2 domain. Upon recruitment to the SSU processome, the N terminus of Dhr1 extends away from the body of the enzyme to engage with the SSU processome, releasing the autoinhibition of the N terminus. In this state, loop 1 remains in the RNA binding cleft and binding to the activation domain of Utp14 is impaired. Displacement of loop 1 favors RNA binding and binding of the activation domain of Utp14. Whether loop 1 is displaced by an accessory factor or by competition with RNA remains to be resolved. However, we conclude that Utp14 does not act by displacing this loop. We note that the autoinhibitory effect of loop 1 may not be universal among Dhr1 proteins, as the SKTTD motif that stabilizes loop 1 in the RNA binding cleft is not conserved. Because mutants of loop 1 designed to disrupt its interaction with the RNA binding cleft enhance Utp14 binding, we propose that the activation domain of Utp14 engages with Dhr1 after the release of loop 1.

In the work presented here, we have used ATPase activity as a proxy for unwinding activity. We previously reported that Dhr1 could unwind a short RNA oligonucleotide bound to U3 snoRNA (14). However, that substrate was not the native substrate for Dhr1 and although the unwinding activity that we observed required ATP, it was not apparently a processive reaction typical of DEAH/RHA family enzymes. Our previous UV crosslinking and genetic studies suggested that the native substrate for Dhr1 is the pair of duplexes formed between U3 snoRNA and two disparate regions of 18S involving the 5′-end and a region more than 1000 nucleotides downstream that come together to form the CPK. More recent structural studies support our earlier conclusions and refine the regions of 18S that basepair with U3 snoRNA as nucleotides 10 to 15 and 1110 to 1121 (9). Based on these findings, we have developed substrates that more closely reflect what we expect to be the native substrate of Dhr1 (33) and which require processive unwinding. Unfortunately, we have not been able to establish robust processive unwinding on these substrates or on general substrates containing 3′-polyA tails either with or without the addition of Utp14. It is possible that additional cofactors are needed to promote the unwinding reaction of Dhr1. For example, Pno1 interacts with Dhr1 in the PostA1 structure. Cryo-EM imaging of reaction intermediates may be required to solve this conundrum.

## Experimental procedures

### Strains and plasmids

All strains are listed in Table 2. Strain AJY4387 was made by homologous recombination of PCR product from pAJ1037 with AJO2958 and AJO2957 and then transformed into AJY3711. Strain AJY3244 was made by homologous recombination of PCR product from pAJ430 with AJO1948 and AJO1949 and intergraded into BY4743. Strain AJY4605 was made by crossing AJY3244 and AJY4387. Strain AJY4605 was made by crossing AJY3244xAJY4387.

**Table 2:** Strains used in this work

Strains	Description	Source
AJY1134	*334 (MATalpha pep4–3 prb1–1122 ura3–52 leu2–3*, *112 reg1–501 gal1)*	(34)
AJY3244	*KanMX-GAL1-3xHA-UTP14 his3Δ1 leu2Δ0 met15Δ0 ura3Δ0*	(31)
AJY3447	*dhr1-gfp his3Δ1 leu2Δ0 met15Δ0 ura3Δ0*	(30)
AJY3711	*MATa KanMX-P*_*GAL*1_*-3xHA-DHR1 his3Δ1 leu2Δ0 met15Δ0 ura3Δ0*	(14)
AJY4387	*CloNAT-P*_*GAL1*_*-3xHA-DHR1*	(31)
AJY4605	*KanMX-P*_*GAL1*_*-3HA-UTP14 Nat-*_*PGAL1*_*-3HA-DHR1 his3Δ1 leu2Δ0 met15Δ0 ura3Δ0*	(31)
AJE454	*Dh5α*	
AJE854	*Codon*^*+*^ *BL21 (DE3) RIL*	

All plasmids are listed in Table 3. FL-Dhr1-WT (pAJ4911) and FL-Dhr1-ΔSKTTD (pAJ5228) were cloned by Golden Gate Assembly into the vector in frame with an amino-terminal MBP-10xHistidine cleavable using tobacco etch virus protease under the control of the galactose-inducible *GAL1* promoter. ΔN-Dhr1-WT (pAJ3487), ΔN-Dhr1-ΔSKTTD (pAJ5222), and ΔN-Dhr1-RecA2 (pAJ5269) surface mutants were cloned by inverse PCR from vector pAJ3082, pAJ 5223, and pAJ 5244, and digestion with NdeI and XhoI ligation into pET21a(+) with a T7 promoter at the N terminus and Hisx6 tag at C terminus. Utp14-A (pAJ4551) and Utp14-G (pAJ4552) were cloned by PCR from pAJ1919 and digestion with BamHI, SalI ligation into the vector in frame with an amino terminal MBP-10xHistidine cleavable using tobacco etch virus protease under the control of the Tac promoter.

**Table 3:** Plasmids used in this work

Plasmid	Description	Source
pRS415	*LEU2 CEN ARS*	
pAJ1919	*UTP14 URA3 CEN ARS*	(25)
pAJ2312	*T7-DHR1-HIS6* in *pET-21a*	(14)
pAJ3264	*utp14-V754G/I755T/E757G/A758G/A760P URA3 CEN ARS*	(25)
pAJ3082	*DHR1 LEU2 CEN ARS*	(14)
pAJ3487	*T7-dhr1-Δ1–375 (ΔN)-HIS6* in pET-21a	This study
pAJ4111	*T7-dhr1-Δ1–375 (ΔN)-R563M-HIS6* in pET-21a	This study
pAJ4113	*T7-dhr1-Δ1–375 (ΔN)-E1037K-HIS6* in pET-21a	This study
pAJ4141	*dhr1-Δ645–718 (Δ74) LEU2 CEN ARS*	This study
pAJ4153	*dhr1-R563M LEU2 CEN ARS*	(31)
pAJ4158	*T7-dhr1-Δ1–375 (ΔN)-F837L-HIS6* in pET-21a	This study
pAJ4162	*T7-dhr1-Δ1–375 (ΔN)-D566Y-HIS6* in pET-21a	This study
pAJ4523	*dhr1-Δ947–983 LEU2 CEN ARS*	This study
pAJ4551	*MBP-HIS6-TEV-utp14A(aa710–779) in pMALc2H10T*	This study
pAJ4552	*MBP-HIS6-TEV-utp14G(aa710–800) in pMALc2H10T*	This study
pAJ4664	*dhr1-E360K LEU2 CEN ARS*	(31)
pAJ4665	*dhr1-E402G LEU2 CEN ARS*	(31)
pAJ4911	*P*_*GAL1*_*-MBP-HIS10-TEV-DHR1*	(33)
pAJ4667	*dhr1-D566Y LEU2 CEN ARS*	(31)
pAJ4670	*dhr1-F837L LEU2 CEN ARS*	(31)
pAJ4671	*dhr1-E1037K LEU2 CEN ARS*	(31)
pAJ5201	*T7-pGEMsnR17A (U3 gene)* in pUC19	(14)
pAJ5222	*T7-dhr1-Δ1–375 (ΔN)-ΔSKTTD-HIS6* in pET-21a	This study
pAJ5223	*dhr1-ΔSKTTD LEU2 CEN ARS*	This study
pAJ5228	*P*_*GAL1*_*-MBP-HIS10-TEV-dhr1-ΔSKTTD*	This study
pAJ5229	T7-pGEMsnR17A (U3gene)_26_UCUAU_30_ to _26_AAUA_29_	This study
pAJ5238	*dhr1-Δ1–375 (ΔN) LEU2 CEN ARS*	This study
pAJ5239	*dhr1-Δ1–375 (ΔN)-ΔSKTTD LEU2 CEN ARS*	This study
pAJ5240	*T7-dhr1-ΔSKTTD-HIS6* in pET-21a	This study
pAJ5244	*dhr1-H593Y/R596C/E831K(YCK) LEU2 CEN ARS*	This study
pAJ5246	*dhr1-E360K/E397D/E402G/D408Y (KDGY) LEU2 CEN ARS*	This study
pAJ5267	*T7-dhr1-Δ1–375 (ΔN)-H593Y/R596C/E831K (YCK)-HIS6* in pET-21a	This study
pAJ5269	*T7-dhr1-Δ1–375 (ΔN)-E397D/E402G/D408Y (DGY)-HIS6* in pET-21a	This study
pAJ5272	*dhr1-Δ656–680 (Δ25) LEU2 CEN ARS*	This study
pAJ5273	*dhr1-Δ651–685 (Δ35) LEU2 CEN ARS*	This study
pAJ5274	*dhr1-Δ646–690 (Δ45) LEU2 CEN ARS*	This study
pAJ5275	*dhr1-Δ666–675 (Δ11) LEU2 CEN ARS*	This study
pAJ5281	*dhr1-E360K/E397D/E402G/D408Y/H593Y/R596C/E831K LEU2 CEN ARS*	This study
pAJ5289	*dhr1-Δ671–676 (ΔEAEDID) LEU2 CEN ARS*	This study
pAJ5293	*dhr1-H593Y LEU2 CEN ARS*	This study
pAJ5294	*dhr1-R596C LEU2 CEN ARS*	This study
pAJ5295	*dhr1-E831K LEU2 CEN ARS*	This study
pAJ5296	*dhr1-E397D LEU2 CEN ARS*	This study
pAJ5297	*dhr1-D408Y LEU2 CEN ARS*	This study

TEV, tobacco etch virus.

### Assay for genetic interactions between Dhr1 and Utp14 mutants

Doubly conditional yeast cells (AJY4605) in which both *DHR1* and *UTP14* were under control of the glucose-repressible *GAL1* promoter were transformed with either a vector for *UTP14* (pAJ1919) or mutant *utp14* (pAJ3264), and with either an empty vector (pRS415), a vector for WT *DHR1* (pAJ3082), or a vector for *mutant dhr1* (plasmids listed in Table 3). Subsequently, 10-fold serial dilutions were performed, and the cells were spotted on synthetic dropout ura-leu- media containing glucose or galactose and grown for 2 days at 30 °C.

### Western blot analysis

Primary antibodies used in this study were rabbit anti-Dhr1 (our laboratory) and rabbit anti-glucose-6-phosphate dehydrogenase (Sigma ImmunoChemicals). Anti-Dhr1 detects a protein of the expected molecular weight which shifts as expected when fused to GFP or the tandem affinity purification tag. Anti-glucose-6-phosphate dehydrogenase detects a protein of the expected molecular weight. Secondary antibody was goat anti-rabbit antibody-IRDye 680RD (Li-Cor Biosciences). Blots were imaged with an Odyssey CLx infrared imaging system (Li-Cor Biosciences) using Image Studio (https://www.licorbio.com.image-studio).

### Protein expression and purification from yeast

For proteins purified from yeast, 1.5 L SD-Leu medium was inoculated with a fresh culture of AJY1134 containing the desired expression plasmid to give a starting *A*_600_ around 0.1. After 6 to 7 h the *A*_600_ reached 0.3 to 0.4, and 75 ml of 20% galactose was added to make a final concentration of 1% for induction overnight (∼16 h) at 30 °C. The next morning, yeast cells were harvested by centrifugation at 4000g for 5 min. The cells were washed with 5 ml cold lysis buffer-Y (50 mM Tris–HCl pH 7.5, 500 mM NaCl, 10% glycerol, 1 mM EDTA, 1 mM DTT, 1 mM PMSF, 1 μM pepstatin A, 1 μM leupeptin, and 1 mM benzamidine) and then resuspended in 0.3 ml lysis buffer-Y per gram of cell pellet. The cell suspension was dripped from a serological pipette into liquid nitrogen in a conical tube to make “popcorn”. The “popcorn” was ground cryogenically in a CryoMill (Retsch MM300) with shaking for 3 min at 15 Hz, repeated 5 times and the “ground sample” was stored at −80 °C. For purification, all steps were performed at 0 to 4 °C. The ground sample was thawed on ice and an equal volume of cold lysis buffer-Y was added. The suspension was clarified by centrifugation at 5000g for 10 min, and the supernatant was subjected to a second round of clarification at 50,000g for 20 min. The clarified extract was processed with amylose affinity chromatography and metal affinity chromatography as described (33).

### Protein expression and purification from *E*.*coli*

ΔN-Dhr1-WT (pAJ3487), ΔN-Dhr1-ΔSKTTD (pAJ5222), and ΔN-Dhr1-RecA2 (pAJ5246) were transformed into *E*.*coli* expression strain AJE854. A fresh 10 ml culture in LB with 75 μg/ml ampicillin and 25 μg/ml chloramphenicol was transferred into 1 L of the same medium and incubated at 37 °C with shaking. At *A*_600_ of 0.6 protein expression was induced with 0.5 mM IPTG overnight (∼16 h) at 16 °C. All subsequent steps were performed at 0 to 4 °C. Cells were harvested and washed with 5 ml lysis buffer-E (40 mM Tris–HCl pH 8, 500 mM NaCl, 10% glycerol, 5 mM β-mercaptoethanol (BME), 1 mM PMSF, 1 μM pepstatin A, 1 μM leupeptin, and 1 mM benzamidine) and resuspended in 1 ml lysis buffer-E per gram of cell pellet. Lysozyme was added to a final concentration of 0.1 mg/ml, and the cell resuspension was incubated on ice for 30 min. The cell suspension was sonication using a Branson Sonifier 250 at: 50% duty, output control at 7, 7 cycles of 20 s pulses with a 2 min rest between each cycle. DNase I, RNase I, and RNase A were added to make a final concentration at 0.1 mg/ml, 10 U/ml, and 2.5 U/ml, respectively, and incubated on ice for 30 min. The suspension was clarified by centrifugation at 5000*g* for 10 min, and the supernatant was subjected to a second round of clarification by centrifugation at 50,000*g* for 20 min. Further steps were carried out using metal affinity chromatography (HisTrap FF) and ion exchange chromatography (HiTrap Capto SP) as described (33).

Utp14-A and Utp14-G purification: pAJ4551 (Utp14-A) and pAJ4552 (Utp14-G) were transformed into AJE854. Cells were cultured in 250 ml LB containing 0.2% glucose and with 75 μg/ml ampicillin and 25 μg/ml chloramphenicol at 37 °C with shaking. Protein expression was induced at *A*_600_ 0.4 to 0.5 with 1 mM IPTG for 4 h. Cells were harvested, washed, and resuspended in cold lysis buffer-E without BME (40 mM Tris pH 8.0, 500 mM NaCl, and 10% glycerol, 1 mM PMSF, 1 μM pepstatin A, 1 μM leupeptin, and 1 mM benzamidine). Cells were lysed by adding 0.1 mg/ml of lysozyme for 30 min at 4 °C, followed by sonication (50% duty cycle, output set at 5, 10 pulses lasting 2 s each, repeated 5 times). The lysate was clarified at 25,000*g* for 20 min at 4 °C and applied to a 1 ml HisTrap HP column (GE HealthCare). The nickel column was washed with lysis buffer-E containing 10 mM imidazole followed by a wash with lysis buffer-E containing 25 mM imidazole. Utp14-A or Utp14-G was then eluted using lysis buffer-E containing 250 mM imidazole. Peak fractions containing Utp14-A or G were pooled and applied to a Q column (HiTrap Q HP anion exchange chromatography column, 1 ml, Cytiva), which was washed with a gradient from 100 mM NaCl to 1 M NaCl in Lysis buffer-E without BME (40 mM Tris pH 8.0, NaCl, and 10% glycerol). Protein purification was assessed by SDS-PAGE, and the cleanest fractions were combined and concentrated by centrifugal filter units, 30kD molecular weight cutoff, sample volume 4 ml (Millipore). All the protein concentrations were determined using Qubit Protein Broad Range (BR) Assay, and the proteins were flash-frozen and stored at −80 °C.

### ATPase assay

The ATPase activities of Dhr1 variants in the presence and absence of Utp14-A or Utp14-G fragments were measured by adapting the EnzChek Pyrophosphate Assay Kit (Thermo Fisher Scientific) at 26.3 °C to 384 well plates to minimize protein consumption. All reactions were performed in triplicate in a buffer containing 40 mM Tris–HCl pH 8.0, 40 mM KCl, 150 mM NaCl, 2 mM NaOAc, 2 mM DTT, 10 mM MgCl_2_, and 4% glycerol in a total volume of 20 μl. Reactions were supplemented with 0.2 U PNP and 1 mM 2-amino-6-mercapto-7-methylpurine ribonucleoside. For all reactions, 0.5 μM Dhr1 (and variants) was used. For reactions carried out in the presence of RNA, RNA concentration was defined by the concentration of nucleotide and the samples were preincubated for 10 min at room temperature. All reactions were initiated by adding ATP and MgCl_2_ to the reactions to make a final concentration of 2 mM. Absorbance at 360 nm was measured every 20 s over 40 min in a microplate reader (Synergy HT multimode reader, BioTek Instruments, Inc; Microplate, 384 well, PS, F-Bottom, clear, nonbinding). To quantify the released Pi, a calibration curve was generated with the phosphate standard provided with the kit. The resulting values were plotted as a function of time using the GraphPad Prism software (https://graphpad.com). To compare ATPase activities between samples, data points within the linear range were fitted with linear regression (33).The data represent mean values from three replicates, while the error bars indicate the standard error.

### RNA binding assay

RNA binding affinities of Dhr1 proteins were determined by FP measurements with 5′-6-FAM-labled U_20_ at 25 °C using CLARIOstar Plus (BMG LABTECH) fluorescence plate reader. All reactions were carried out in triplicate in a buffer containing 40 mM Tris–HCl pH 8.0, 40 mM KCl, 150 mM NaCl, 2 mM NaOAc, 2 mM DTT, 10 mM MgCl_2_, 4% glycerol, and 0.05% Tween 20 in a total volume of 20 μl. For the RNA binding measurements, 1.25 nM 5′-6-FAM-labeled U_20_ RNA was applied in all reactions, ΔN-Dhr1-WT or ΔN-Dhr1-ΔSKTTD were applied at varying concentrations ranging from 0.625 nM to 20 μM. For measurements performed in the presence of adenosine nucleotides, ADP, and AMPPNP (Roche) were added at final concentrations of 1 mM. Reactions were incubated at 25 °C for 10 min and then read by the plate reader.

### Peptide binding assay

The binding affinities of Dhr1 variants for Utp14-A′ were determined by FP measurements with 5′-6-FAM-labled peptides (UTP14 746–780: VFMFKQQDVIAEAFAGDDVVAEFQEEKKRVIDDED, Genscript) at 25 °C using CLARIOstar Plus (BMG Labtech) fluorescence plate reader. All reactions were carried out in triplicate in a buffer containing 40 mM Tris–HCl pH 8.0, 40 mM KCl, 150 mM NaCl, 2 mM NaOAc, 2 mM DTT, 10 mM MgCl_2_, 4% glycerol, and 0.05% Tween 20 in a total volume of 20 μl. For the RNA binding measurements, 1.25 nM 5′-6-FAM-labeled Utp14-A′ was titrated ΔN-Dhr1-WT or ΔN-Dhr1-ΔSKTTD or ΔN-Dhr1-YCK (RecA2 mutants) at varying concentrations ranging from 0.625 nM to 20 μM. For measurements performed in the presence of adenosine nucleotides, ADP and AMPPNP (Roche) were added at final concentrations of 1 mM. Reactions were incubated at 25 °C for 10 min and then read by the plate reader.

### Dhr1-Utp14 interactions detection by mass photometry

The MP experiments were carried out on a TwoMP instrument (Refeyn) at room temperature (25.3 °C). Recommended standards, beta-amylase and thyroglobulin (bovine) were purchased from Refeyn. Standards were diluted with PBS buffer to a final concentration of 50 nM. Sample preparation Kit was purchased from Refeyn. Briefly, 17 μl of buffer containing 40 mM Tris–HCl pH 8.0, 40 mM KCl, 150 mM NaCl, 2 mM NaOAc, 2 mM DTT, 10 mM MgCl_2_, and 4% glycerol was mixed with 3 μl of 50 nM standard stock on the sample surface. Reasonable counts (>3000) and relative molecular weight signals were generated (Fig. S11, *A*and *B*). Apo proteins were also diluted in the same buffer and generated relative signals (Fig. S11, *C*–*F*). Data analysis and figure preparation were done directly in the associated program Refeyn AcquireMP (https://refeyn.com/software-release-updates#AcquireMP).

## Data availability

All data are contained within the manuscript.

## Supporting information

This article contains supporting information.

## Conflict of interest

The authors declare that they have no conflicts of interest with the contents of this article.

## Supporting information

Supplementary Martials
