# Supplementary material for: In vitro characterization of the yeast DEAH/RHA RNA helicase Dhr1
Source: J Biol Chem. 2025 Feb 28;301(4):108366. doi: 10.1016/j.jbc.2025.108366 (PMC11994318; doi:10.1016/j.jbc.2025.108366)
Supplement: Supplementary Martials [file mmc1.docx]

**Supplemental Material Figure Legend**

Figure S1. The structural snapshots of the Dhr1 during SSU Processome progression. The yeast SSU Processome (left) is from PDB#7AJU, the Dis-C complex (middle) is from PDB#6ZQG, and the early pre-40S (right) is from humans (PDB#6G4W). Dhr1(pink) is shown in surface density in the Processome and Dis-C complex.

Figure S2. (A). A schematic of Dhr1 domain organization with multi-sequence alignment of Loop1 region of Dhr1 RecA2 domain and its analogs. SKTTD motif was highlighted in purple. The sequences have been aligned using T-coffee^7^ and adjusted manually then output using ESPript 3.0.^8^ Aligned sequences: protein, organism (access No.): Dhr1, *S. cerevisiae* S288C (NP_013847.1); AFR222Wp, *E. gossypii* ATCC 10895 (NP_985769.2); Dhr1 (*K.unispora*, KAG0662609.1); Dhr1, *S. pombe* (NP_593520.1); Rha-2, *C.elegans*

(NP_498895.1); DHX37, *X. tropicalis* (XP_002944206.3); DHX37 *D.rerio* (NP_001076473.2); DHX37, *Mus musculus* (NP_976064.1); DHX37 *H. sapiens* (NP_116045.2) (B). Dhr1 complementation. *P_GAL1_-DHR1* (AJY3711) cells containing either empty vector (EV), wild type (WT) or vectors encoding the indicated alleles of *DHR1* spotted on SD-Leu containing glucose (left) or galactose (right) grown for 48 hours at 30ºC. (C). To determinie if Dhr1 mutants were expressed comparably to wild-type Dhr1, the indicated Dhr1 mutants were expressed in AJY3447 (GFP-tagged Dhr1) and expression was monitored by western blotting for Dhr1.The positions of molecular weight markers, genomically expressed Dhr1-GFP as well as plasmid expressed Dhr1 mutants and G6PD as a loading control are shown.

Figure S3. RNA binding affinity of Dhr1 and mutant variants. Fluorescence-polarization equilibrium binding assays of ∆N-Dhr1-WT or the ∆N-Dhr1-∆SKTTD. 5’ 6-FAM fluorophore-labeled U20 RNA in the absence of adenosine nucleotides (A) or with 1 mM ADP (B) or with 1 mM AMPPNP (C). Bmax and Kd values are provided in (D).

Figure S4. *P*_GAL1_*-UTP14* *P_GAL1_-DHR1* (AJY4605) cells containing either WT *UTP14* (UTP14-WT)(left) or Mutant *UTP14* (UTP14-M)(right), WT *DHR1* or vectors encoding the indicated alleles of *DHR1* were transformed into these two different strains with spotted on SD-Leu-Ura containing grown for 48 hours at 30ºC.

Figure S5. Intrinsic ATPase activity of different Dhr1s with the presence of Utp14-A or Utp14-G. 0.5 µM FL-Dhr1-WT (A) or FL-Dhr1-∆SKTTD (B) or ∆N-Dhr1-WT (C) or ∆N-Dhr1-∆SKTTD (D) was used in each reaction. Assays were performed in triplicates. Data points represent the mean of the triplicates, and the error bars represent the standard error of the means. 2 µM of Utp14-A or Utp14-G were used.

Figure S6. PolyA -stimulated ATPase activity of Dhr1 variants in the presence and absence of Utp14-A. ATPase activities were measured at various polyA concentrartions (0-100 µM, concentration of single adenosine). Assays were performed in triplicates. Data points represent the mean of triplicates, and error bars represent the standard error of the means. In all assays, 0.5 µM of the Dhr1 was used, and 2 µM of Utp-A was used.

Figure S7. PolyA -stimulated ATPase activity of Dhr1 variants in the presence and absence of Utp14-G. ATPase activities were measured at various polyA concentrartions (0-100 µM, concentration of single adenosine). Assays were performed in triplicates. Data points represent the mean of triplicates, and error bars represent the standard error of the means. In all assays, 0.5 µM of the Dhr1 was used, and 2 µM of Utp-G was used.

Figure S8. (A). Primary structure of Dhr1 displaying domains. All Dhr1 mutations detected in the large-scale genetic screen (Figure 3A) are also displayed (salmon). (B)**.** Crystal structure of yeast Dhr1 (PDB: 7MQJ), with each domain displayed in the same color pattern as in the primary structure in Figure 1A. Mutations of Dhr1 that were selected for further genetics and biochemical analysis are shown. (C). *P_GAL1_-UTP14* *P_GAL1_-DHR1* (AJY4605) cells containing either WT *UTP14* (UTP14-WT) (left) or Mutant *UTP14* (UTP14M)(right),

WT *DHR1* or vectors encoding the indicated alleles of *DHR1* were transformed into these two different strains with spotted on SD-Leu containing grown for 48 hours at 30ºC.

Figure S9. (A). ATPase activity of ∆N-Dhr1-WT and its mutant variants was measured at various polyA concentrations (0-100 uM). Assays were performed in triplicates(N=3). Data points represent the mean of the triplicates, and the error bars represent the standard error of the means. (B). In all assays, 0.5 µM of the enzyme was used. ATPase rates were normalized to allow direct comparison. The curves were fitted based on Michaelis-

Menten analysis. Kapp and kcat were calculated and presented in (B).

Figure S10. (A). Dhr1 complementation.*P_GAL1_-DHR1* (AJY3711) cells containing either empty vector (EV), wild type (WT) or vectors encoding the indicated alleles of *DHR1* spotted on SD-Leu containing glucose for 24 hours(left) or grown for 48 hours at 30ºC. (B). To confirm the phenotype of Dhr1 surface mutants are not coming from the different expression level, all the surface mutants were transformed in the AJY3447 (GFP-tagged Dhr1). As shown above, all the surface mutants expressed at the endogenous Dhr1 level (lane 1).

Figure S11. (A). Calibration with 10 nM Beta-Amylase. 56 kD (Monomer) and 112 kD (Dimer) are the major components. 56 kD peak is also merged with the buffer peak. (B). Calibration confirmation with 10 nM Thyroglobulin (Bovine), 670 kD (Dimer) is the major compotnent. (C). 10 nM of ∆N-Dhr1-WT. (D). 10 nM of ∆N-Dhr1-∆SKTTD. (E). 10 nM of ∆N-Dhr1-YCK. (F). 10 nM of Utp14-G.
