# Supplementary figures and images for: In vitro characterization of the yeast DEAH/RHA RNA helicase Dhr1
Source: J Biol Chem. 2025 Feb 28;301(4):108366. doi: 10.1016/j.jbc.2025.108366 (PMC11994318; doi:10.1016/j.jbc.2025.108366)

Post-A1\*  
A1 site cut  
Dhr1-ADP(open)

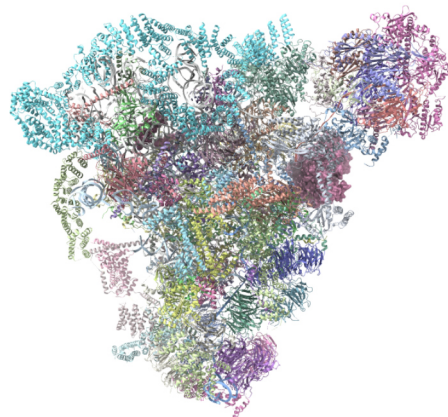

Dis-C  
Dhr1 conformational change  
Dhr1-apo(Close)

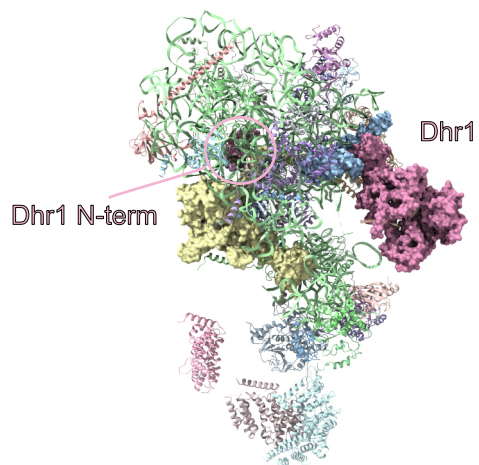

Pre-40S

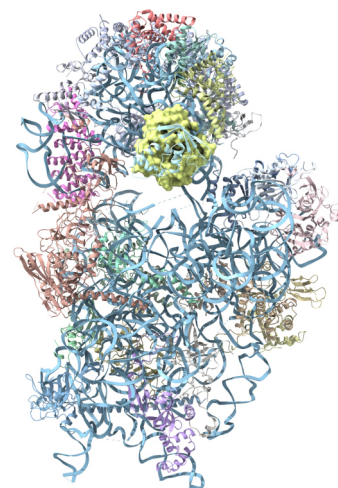

Supplement: Figure S1 [file mmc2.pdf]

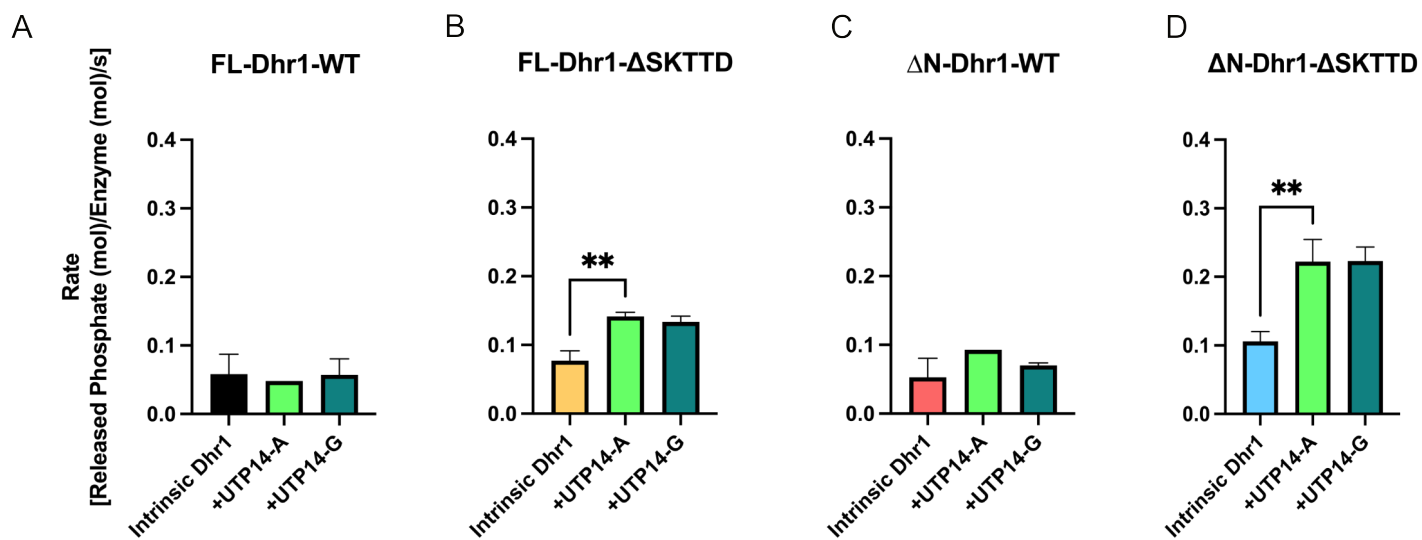

Supplement: Figure S5 [file mmc6.pdf]

A

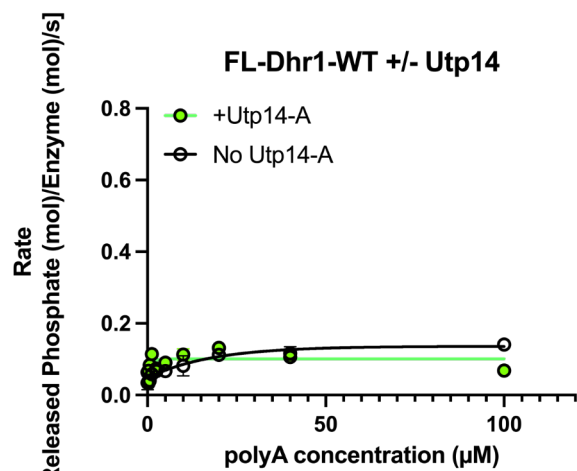

B

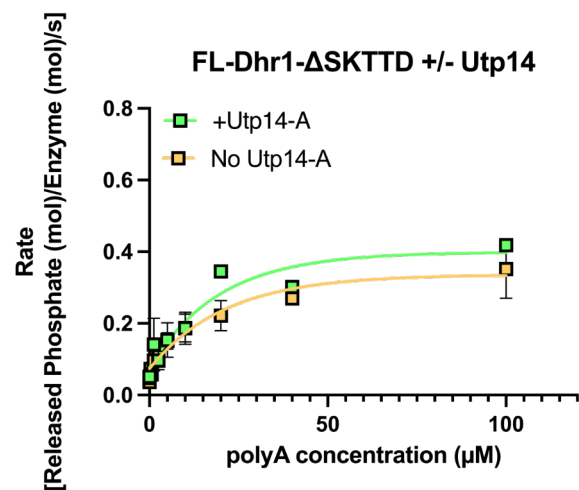

C

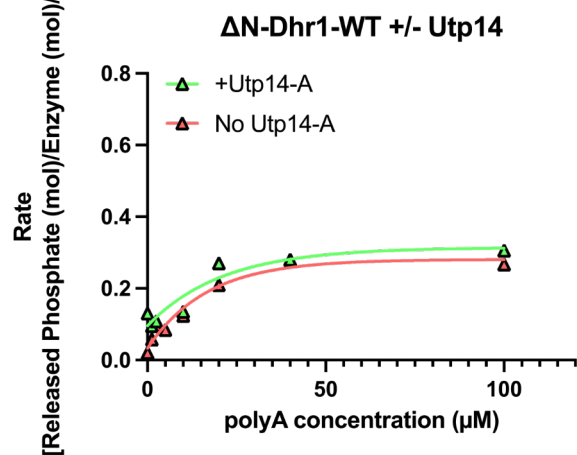

D

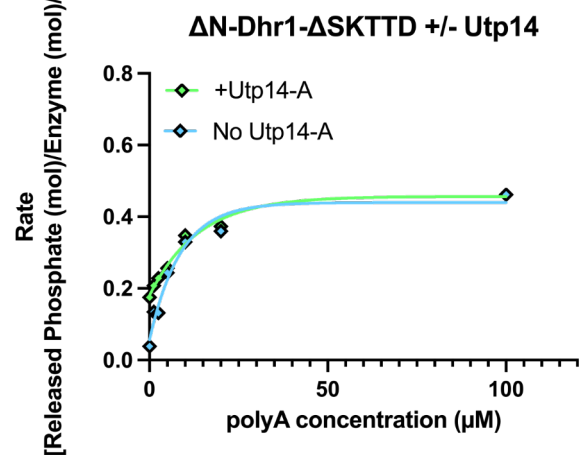

Supplement: Figure S6 [file mmc7.pdf]

A

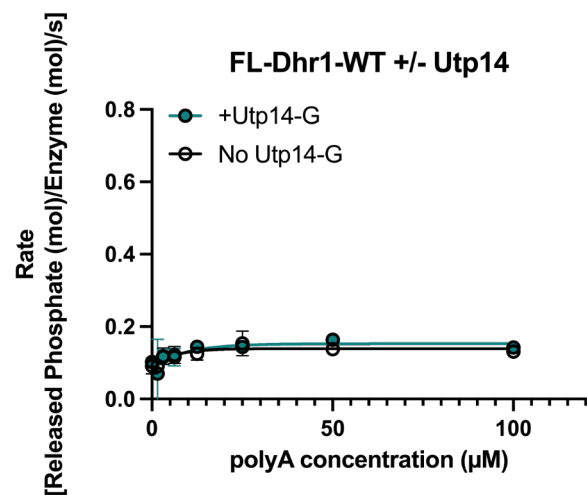

B

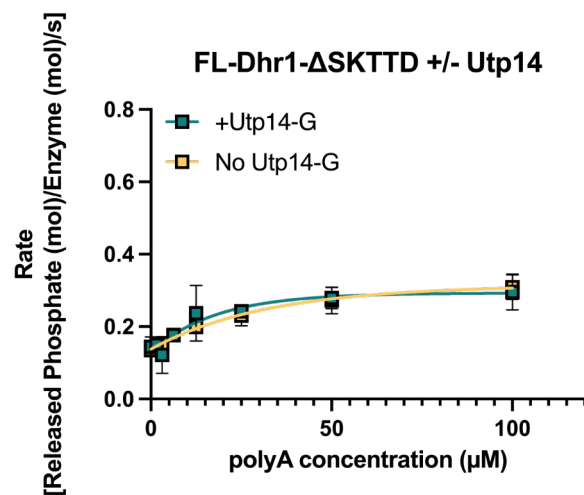

C

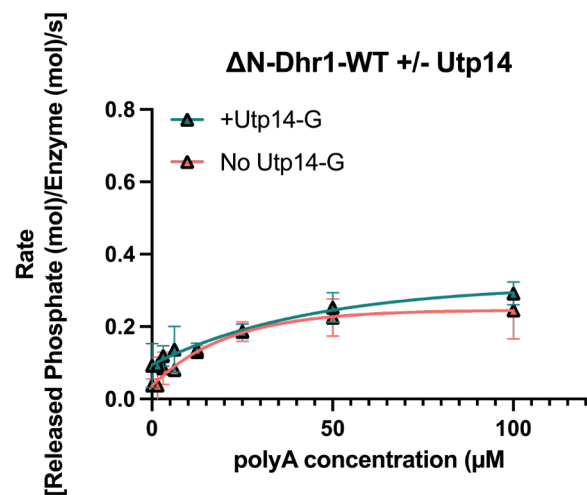

D

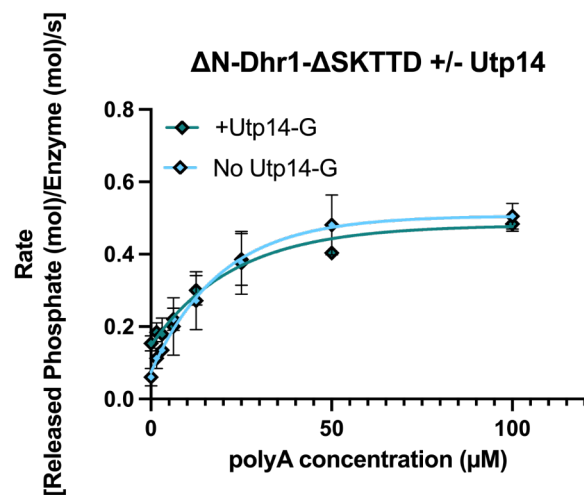

Supplement: Figure S7 [file mmc8.pdf]

A

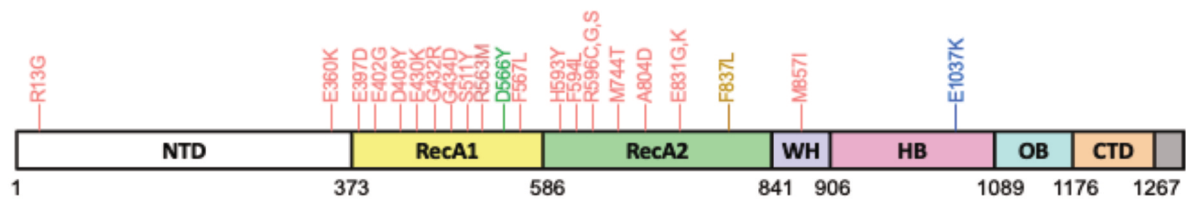

B

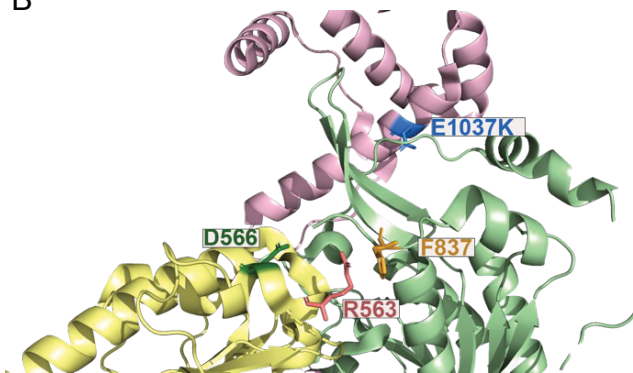

C

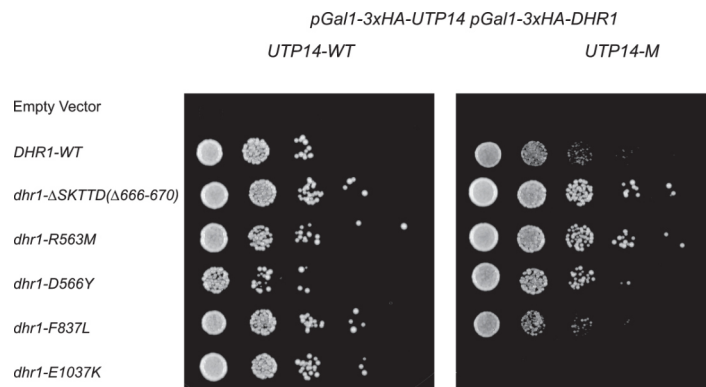

Supplement: Figure S8 [file mmc9.pdf]

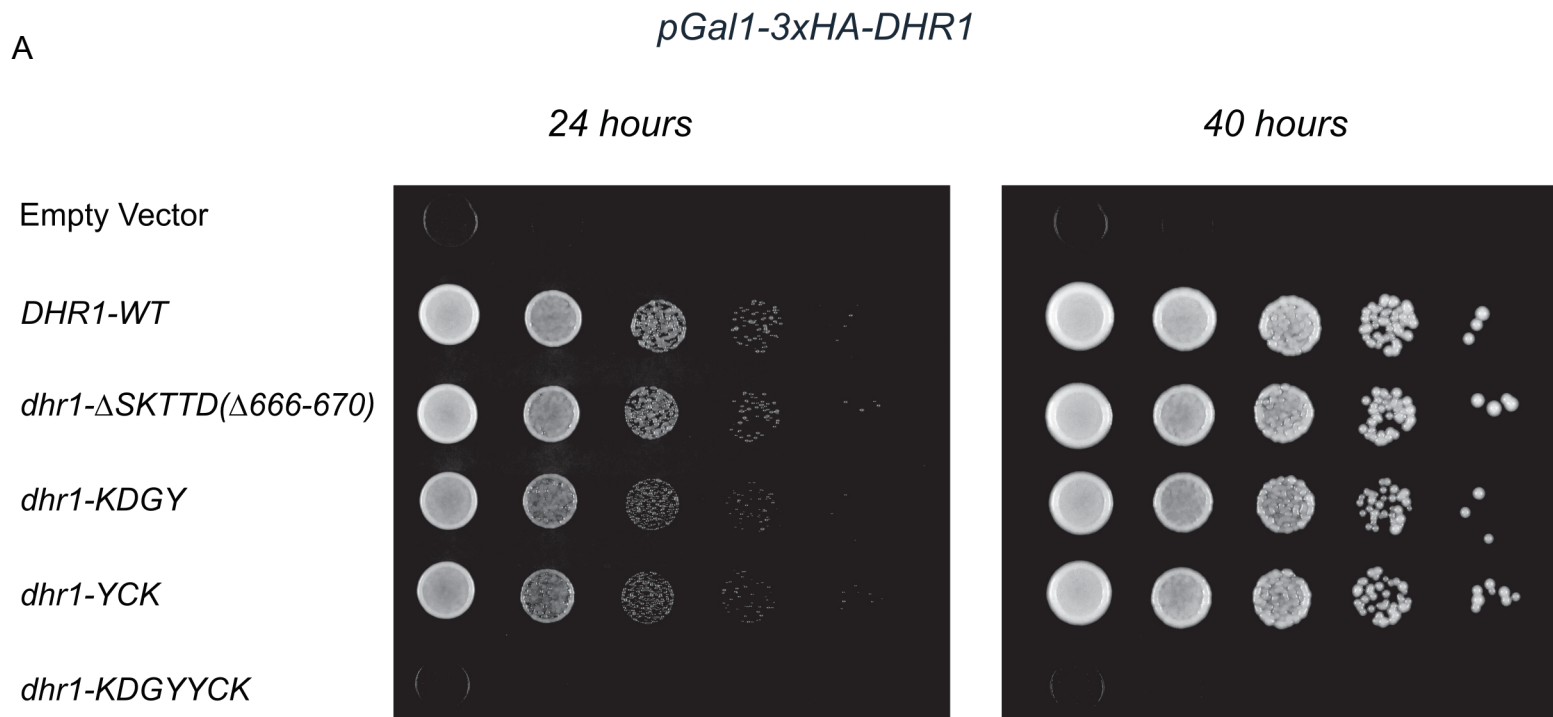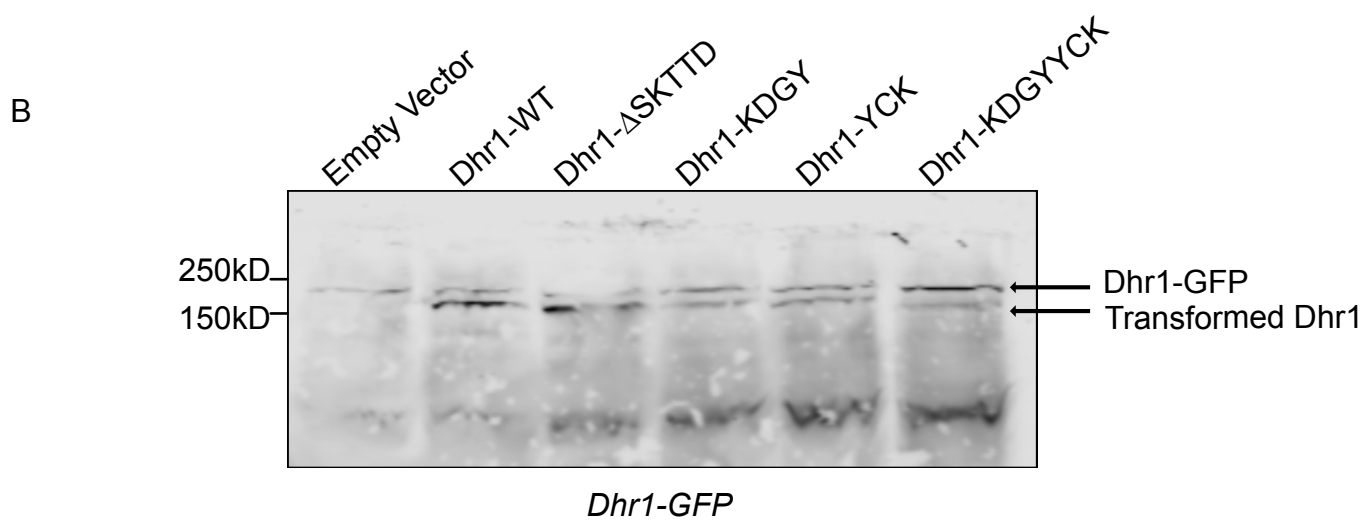

Supplement: Figure S10 [file mmc11.pdf]

A

Calibrantion

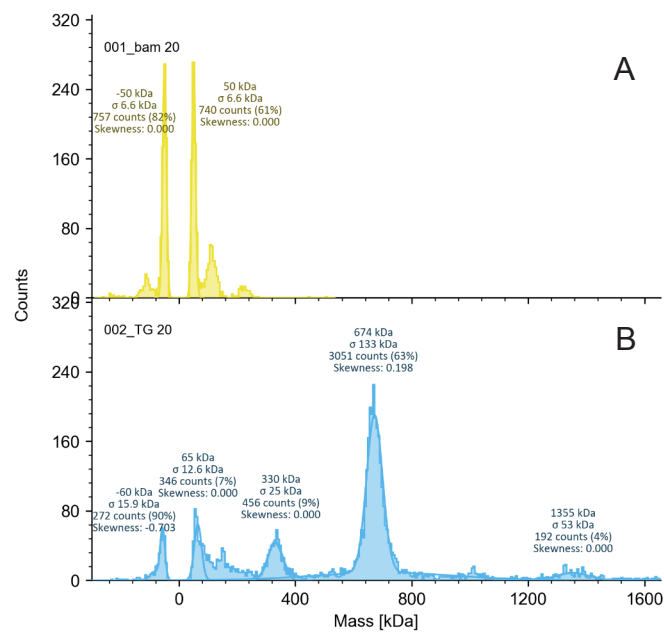

Intrinsic Proteins

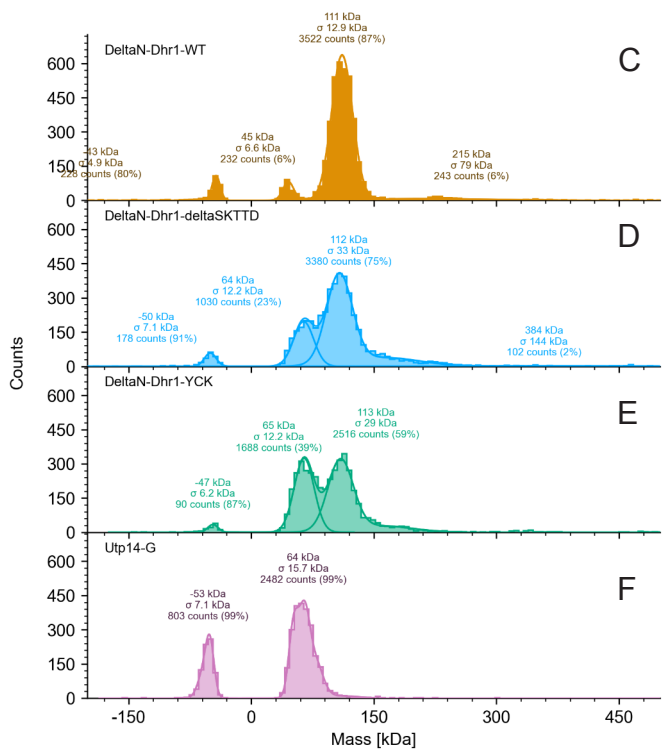

Supplement: Figure S11 [file mmc12.pdf]
