# Supplementary material for: In vitro characterization of the yeast DEAH/RHA RNA helicase Dhr1
Source: J Biol Chem. 2025 Feb 28;301(4):108366. doi: 10.1016/j.jbc.2025.108366 (PMC11994318; doi:10.1016/j.jbc.2025.108366)
Supplement: Figure S2 [file mmc3.pdf]

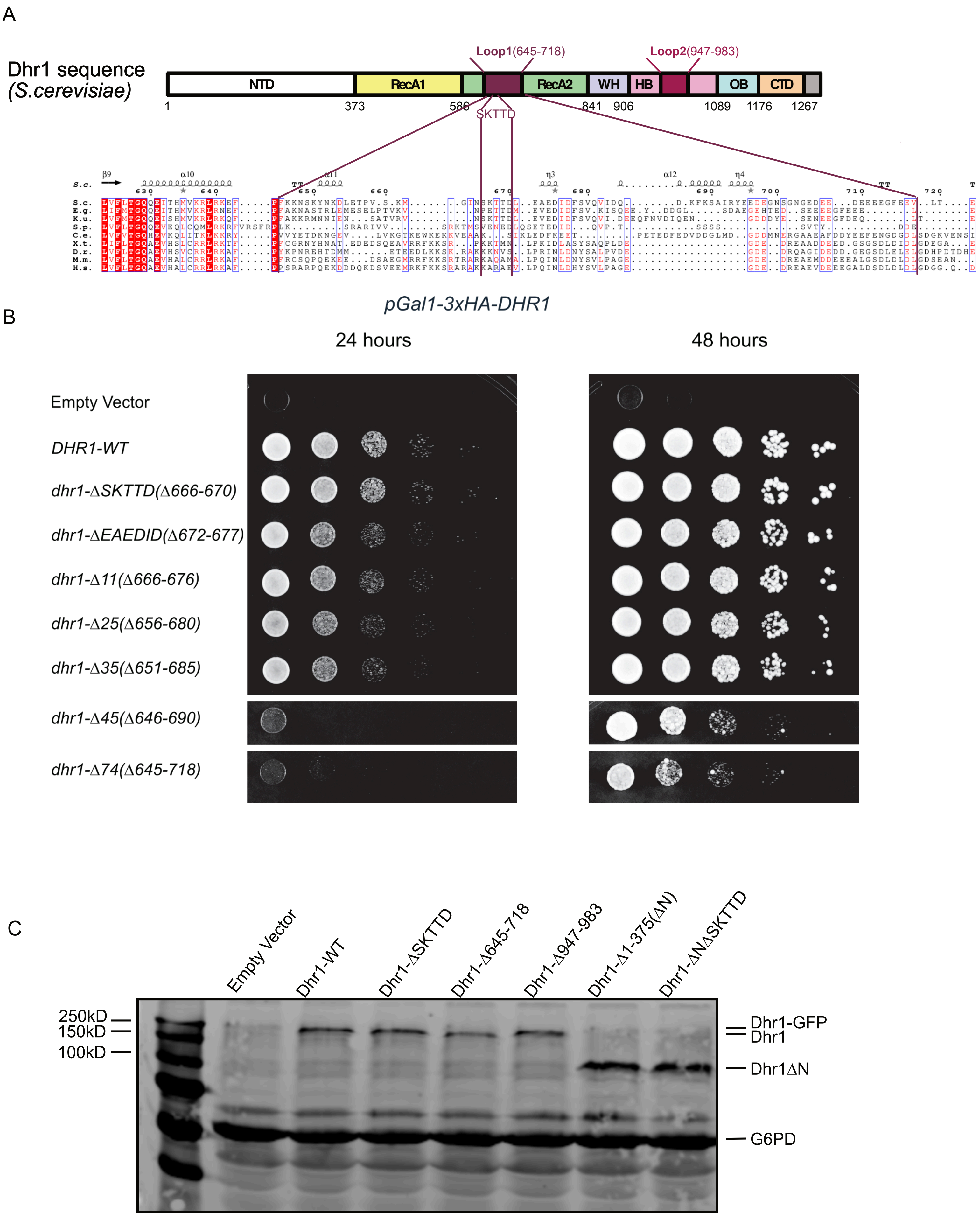

Lin et al Figure S2
