# Supplementary material for: In vitro characterization of the yeast DEAH/RHA RNA helicase Dhr1
Source: J Biol Chem. 2025 Feb 28;301(4):108366. doi: 10.1016/j.jbc.2025.108366 (PMC11994318; doi:10.1016/j.jbc.2025.108366)
Supplement: Figure S3 [file mmc4.pdf]

A

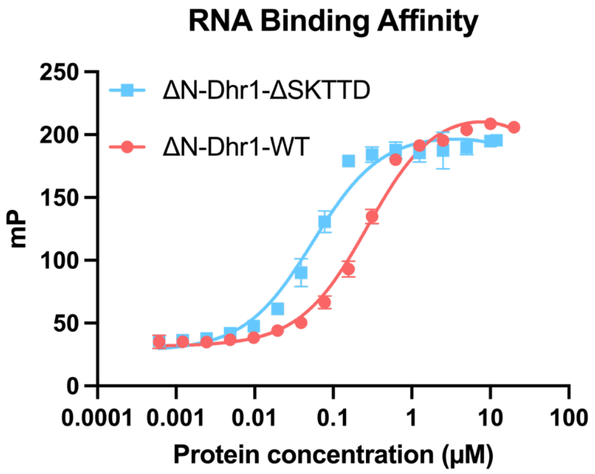

B

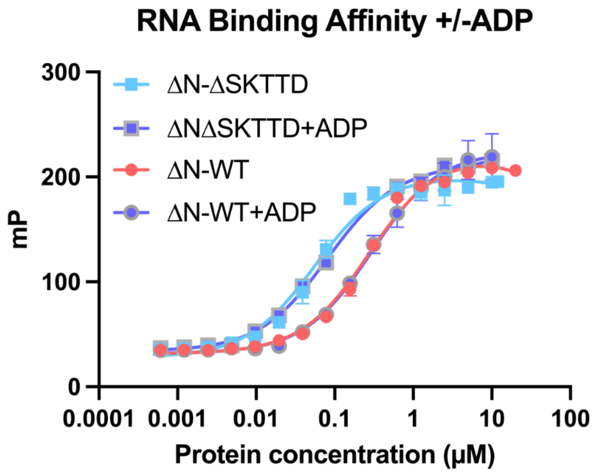

C

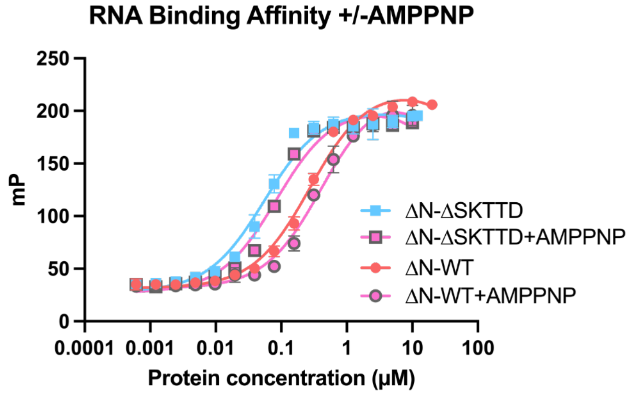

D

RNA binding parameter of Dhr1 and mutant various

	Bmax (mP)	Kd (μM)
**ΔN-Dhr1-WT**	199.63±3.51	0.30±0.02
**ΔN-Dhr1-ΔSKTTD**	174.30±5.20	0.06±0.01
**ΔN-Dhr1-WT+ADP**	193.40±9.30	0.29±0.01
**ΔN-Dhr1-ΔSKTTD+ADP**	175.00	0.08
**ΔN-Dhr1-WT+AMPPNP**	194.40	0.42
**ΔN-Dhr1-ΔSKTTD+AMPPNP**	178.80	0.09

Lin et al Figure S3
