# Supplementary material for: In vitro characterization of the yeast DEAH/RHA RNA helicase Dhr1
Source: J Biol Chem. 2025 Feb 28;301(4):108366. doi: 10.1016/j.jbc.2025.108366 (PMC11994318; doi:10.1016/j.jbc.2025.108366)
Supplement: Figure S9 [file mmc10.pdf]

A

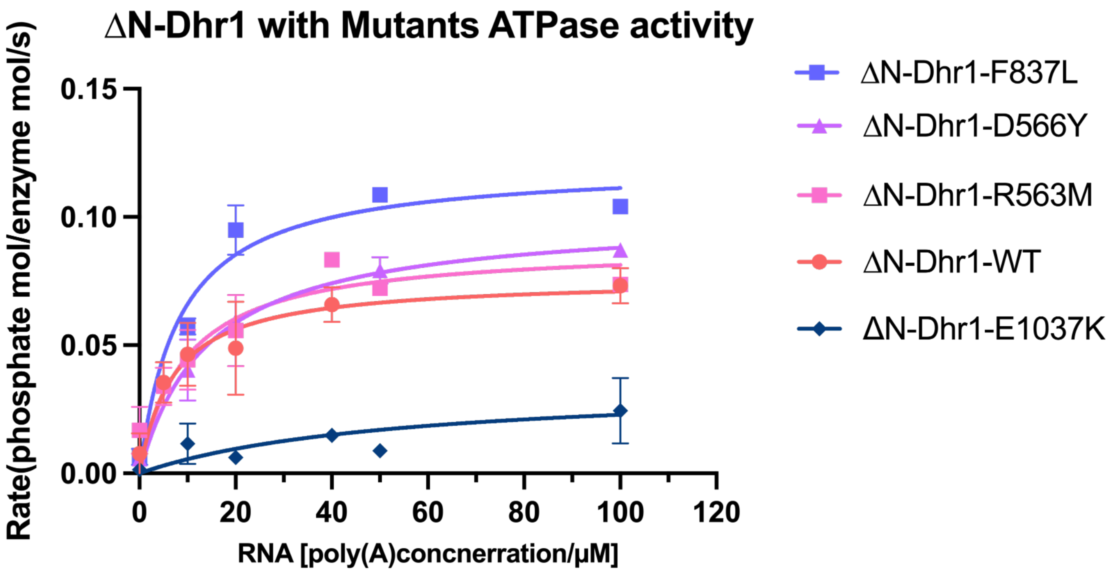

B

Kinetics parameter of ATPase activity of ΔN-Dhr1 and mutant various

	Kapp(μM)	***Vmax***(s^{-1})
**ΔN-Dhr1-WT**	9.81±5.66	0.08±0.01
**ΔN-Dhr1-R563M**	13.44±9.55	0.10±0.03
**ΔN-Dhr1-D566Y**	14.40±4.89	0.10±0.01
**ΔN-Dhr1-F837L**	8.31±0.69	0.12±0.01
**ΔN-Dhr1-E1037k**	61.64±39.49	0.03±0.03

Lin et al Figure S9
